# What has been researched about implicit memory in speech-language-hearing pathology? A scoping review

**DOI:** 10.1590/2317-1782/e20250338en

**Published:** 2026-08-03

**Authors:** Fernanda Roberta de Faria Rocha da Silva, Gilberto da Cruz Leal, Roberta de Carvalho Barabás, Katia Nemr

**Affiliations:** 1 Departamento de Fisioterapia, Fonoaudiologia e Terapia Ocupacional – FOFITO, Faculdade de Medicina, Universidade de São Paulo – USP - São Paulo (SP), Brasil.; 2 Instituto Claretiano de Ensino - Batatais (SP), Brasil.

**Keywords:** Repetition Priming, Implicit Memory, Speech-Language Pathology, Bias, Implicit, Scoping Review

## Abstract

**Purpose:**

to map how implicit memory has been addressed in international scientific speech-language-hearing production.

**Research strategies:**

An electronic search was conducted in the PubMed, Web of Science, Scopus, Embase, CINAHL, and PsycINFO databases, supplemented by a manual search of references of the included studies. The scoping review was conducted based on the Joanna Briggs Institute Evidence Synthesis Manual, following the PRISMA-ScR guidelines. The protocol was registered in the Open Science Framework (10.17605/OSF.IO/PNDWF).

**Selection:**

**criteria**: Studies addressing implicit memory in speech-language-hearing contexts, in Portuguese, English, or Spanish, regardless of year of publication, were included.

**Data:**

**analysis**: Two reviewers independently extracted data, considering design, population, objectives, and main outcomes. The studies were grouped by area of application and synthesized by descriptive and narrative analysis.

**Results:**

A total of 2,565 documents were identified, of which 39 comprised this review. The studies were grouped into three categories (language, audiology, and voice). The most frequent themes found were the application of implicit learning in speech-language-hearing contexts; the use of implicit tasks for the assessment and characterization of disorders; and investigations of biases, prejudices, and/or implicit perceptions in speech-language-hearing practice.

**Conclusion:**

The literature deals with implicit memory in a limited and heterogeneous manner, mainly in language, audiology, and voice, focusing on implicit learning, assessment, and biases. Despite its clinical potential, there is a lack of conceptual and methodological systematization, indicating the need for future research to consolidate its use in the field.

## INTRODUCTION

Around the 1970s, the study of human memory underwent a considerable transformation that, according to Richardson-Klavehn and Bjork^(1)^, revolutionized how we measure and interpret the influence of past events on experience and behavior^(1)^. The central landmark of this change was the recognition that the memory of past experiences can be expressed through two distinct pathways: explicit memory and implicit memory^(2)^.

Explicit memory refers to the conscious recall of recently acquired information. Tests that analyze this type of memory expose individuals to materials (such as words and pictures) and then ask them to consciously recall or recognize these items. Most studies on human memory have focused on this type of process^(2,3)^.

Unlike explicit memory, which is conscious, implicit memory is unconscious, involving content that is not recalled through words, but through performance^(4)^. Implicit memory is assessed through tasks that do not require conscious recall of previous episodes, such as completing words that are presented in a fragmented way, preferring a previously exposed stimulus among a group of stimuli and/or reading inverted texts^(2)^.

Among the manifestations of implicit memory, the most studied phenomenon is priming or repetition, in which prior exposure to a word or object facilitates its recognition or subsequent processing, in the face of minimal perceptual cues^(5)^. In addition to priming, various forms of perceptual, motor, and cognitive learning are also considered expressions of implicit memory, as long as they occur without conscious reference to previous learning episodes. In operational terms, priming is generally observed after a few exposures and is linked to memory for specific items, while skill learning tends to require multiple exposures and does not depend on the recall of specific elements^(2,5)^.

Other manifestations of implicit memory have also been described, including biasing effects of prior experiences on perceptual, cognitive, affective, and social judgments^(2)^. These effects illustrate the breadth of domains in which implicit memory can operate.

Speech-language-hearing (SLH) sciences focus on the study, prevention, evaluation, and treatment of communication disorders such as language, speech, voice, and hearing. Many clinical and therapeutic behaviors in this field involve automatic and unconscious processes. The acquisition and modification of communicative patterns often result from repetitive practices, sensorimotor adjustments, and incidental learning, typical characteristics of implicit processes^(6-11)^.

Although implicit memory is frequently mobilized indirectly in SLH assessment and therapeutic practices, there is a lack of systematization to identify how this construct is conceptualized, operationalized, and empirically applied in the field. It is still unclear which SLH areas have incorporated implicit memory, which designs and tasks have been used to assess it, and which clinical or investigative objectives have guided its use. This gap hinders both the theoretical consolidation of the topic and the translation of the knowledge produced into evidence-based clinical practice. Therefore, this review aimed to map how implicit memory has been addressed in international scientific SLH production.

## METHODS

**Figure 1:**
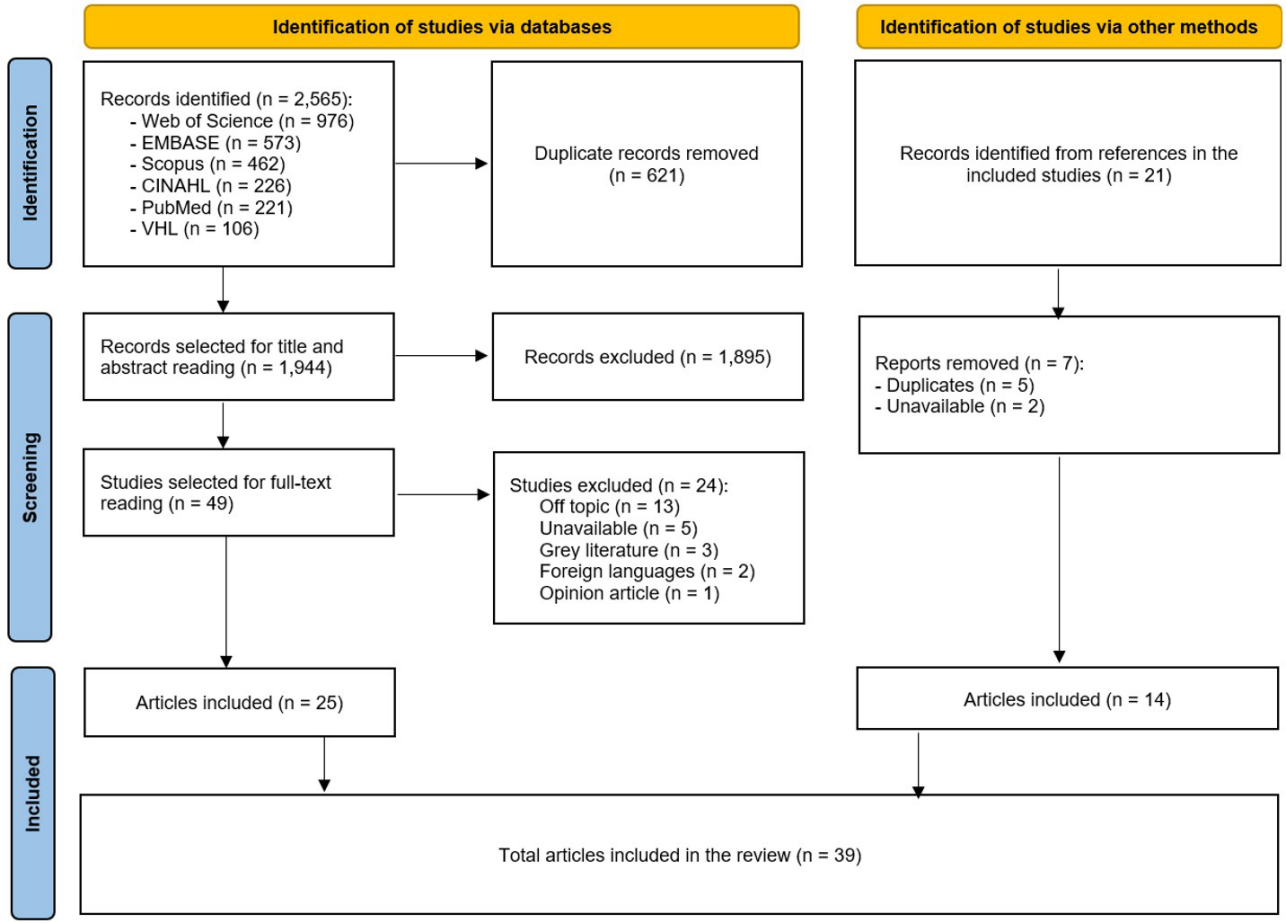
PRISMA Flowchart

Source: Based on Page et al.^(13)^

This is a scoping review based on the Joanna Briggs Institute (JBI) manual^(12)^ and the recommendations of the Preferred Reporting Items for Systematic Reviews and Meta-Analyses – Extension for Scoping Reviews (PRISMA-ScR)^(13)^ (Figure 1). The protocol for this review was registered on the Open Science Framework platform (10.17605/OSF.IO/PNDWF).

The following steps were defined for this study: identification of the research question; establishment of eligibility criteria aligned with the research question and objective; development of the search strategy; identification of relevant studies; selection of studies; data extraction; and data mapping and summarization of the results. The study also assessed the level of evidence of the studies.

### Research question

The following research question was defined based on the acronym PCC (population, concept, and context), where the population is SLH pathology and the concept is implicit memory: "What has been studied about implicit memory in SLH pathology?". The authors chose not to use specific descriptors for the context to broaden the scope of the results.

### Eligibility criteria

The review included studies published in Portuguese, English, and Spanish, without restriction as to the publication period, and excluded studies whose access was conditional on payment or that were not available in full, even after attempts to contact the authors (it should be noted that a significant portion of the journals were accessed free of charge through the institutional network of the University of São Paulo, Brazil). Documents originating from grey literature (such as abstracts from conferences and proceedings, book chapters, dissertations, and theses), as well as letters to the editor, opinion articles, and reviews, were also excluded.

The references in the included studies were analyzed, and those that fit the research topic and the other criteria mentioned above were included.

### Search strategies

The keywords and descriptors used for the search were identified in the Health Sciences Descriptors (DeCS), Medical Subject Headings (MeSH), and previous literature searches (Table 1).

**Table 1:** Descriptors found in automatic and manual searches

Speech-language-hearing pathology	"Voice Quality" OR "Qualities, Voice" OR "Quality, Voice" OR "Voice Qualities" OR "Speech-Language Pathology" OR "Language Pathology" OR "Pathology, Language" OR "Pathology, Speech" OR "Pathology, Speech Language" OR "Pathology, Speech-Language" OR "Speech and Hearing Science" OR "Speech and Hearing Studies" OR "Speech and Hearing Study" OR "Speech and Language Pathology and Audiology" OR "Audiology" OR "Speech Language Pathology" OR "Speech Pathology" OR "Speech, Language and Hearing Pathology" OR "Speech, Language and Hearing Sciences" OR "Speech, Language and Hearing Studies" OR "Speech, Language and Hearing Study" OR "Speech-Language Pathology and Audiology" OR "Speech-Language-Hearing Pathology"
Implicit memory	memory OR memories OR "implicit memory" OR "memory, implicit" OR "Repetition Priming" OR "Priming, Repetition" OR "Bias, Implicit" OR "Bias, Hidden" OR "Bias, Subconscious" OR "Bias, Unconscious" OR "Hidden Bias" OR "Implicit Bias" OR "Subconscious Bias" OR "Unconscious Bias" OR "Implicit association test"

Source: The authors

A new search based on the defined descriptors was conducted in the Medline, Virtual Health Library (VHL), Excerpta Medica Database (EMBASE), Web of Science, SciVerse Scopus (SCOPUS), and Cumulative Index to Nursing and Allied Health Literature (CINAHL) databases on April 15, 2025. The search strategies were adapted for each database using the Boolean operators AND and OR (Table 2).

**Table 2:** Search strategy

Medline	(memory[Text Word] OR memories[Text Word] OR "implicit memory"[Text Word] OR "memory, implicit"[Text Word] OR "Repetition Priming"[Text Word] OR "Priming, Repetition"[Text Word] OR "Bias, Implicit"[Text Word] OR "Bias, Hidden"[Text Word] OR "Bias, Subconscious"[Text Word] OR "Bias, Unconscious"[Text Word] OR "Hidden Bias"[Text Word] OR "Implicit Bias"[Text Word] OR "Subconscious Bias"[Text Word] OR "Unconscious Bias"[Text Word] OR "Implicit association test"[Text Word]) AND ("Voice Quality"[Text Word] OR "Qualities, Voice"[Text Word] OR "Quality, Voice"[Text Word] OR "Voice Qualities"[Text Word] OR "Speech-Language Pathology"[Text Word] OR "Language Pathology"[Text Word] OR "Pathology, Language"[Text Word] OR "Pathology, Speech"[Text Word] OR "Pathology, Speech Language"[Text Word] OR "Pathology, Speech-Language"[Text Word] OR "Speech and Hearing Science"[Text Word] OR "Speech and Hearing Studies"[Text Word] OR "Speech and Hearing Study"[Text Word] OR "Speech and Language Pathology and Audiology"[Text Word] OR "Audiology"[Text Word] OR "Speech Language Pathology"[Text Word] OR "Speech Pathology"[Text Word] OR "Speech, Language and Hearing Pathology"[Text Word] OR "Speech, Language and Hearing Sciences"[Text Word] OR "Speech, Language and Hearing Studies"[Text Word] OR "Speech, Language and Hearing Study"[Text Word] OR "Speech-Language Pathology and Audiology"[Text Word] OR "Speech-Language-Hearing Pathology"[Text Word])
VHL	(memory OR memories OR "implicit memory" OR "memory, implicit" OR "Repetition Priming" OR "Priming, Repetition" OR "Bias, Implicit" OR "Bias, Hidden" OR "Bias, Subconscious" OR "Bias, Unconscious" OR "Hidden Bias" OR "Implicit Bias" OR "Subconscious Bias" OR "Unconscious Bias" OR "Implicit association test" ) AND ("Voice Quality" OR "Qualities, Voice" OR "Quality, Voice" OR "Voice Qualities" OR "Speech-Language Pathology" OR "Language Pathology" OR "Pathology, Language" OR "Pathology, Speech" OR "Pathology, Speech Language" OR "Pathology, Speech-Language" OR "Speech and Hearing Science" OR "Speech and Hearing Studies" OR "Speech and Hearing Study" OR "Speech and Language Pathology and Audiology" OR "Audiology" OR "Speech Language Pathology" OR "Speech Pathology" OR "Speech, Language and Hearing Pathology" OR "Speech, Language and Hearing Sciences" OR "Speech, Language and Hearing Studies" OR "Speech, Language and Hearing Study" OR "Speech-Language Pathology and Audiology" OR "Speech-Language-Hearing Pathology") AND NOT db:("MEDLINE") AND instance:"regional" AND instance:"regional"
EMBASE	(memory OR memories OR 'implicit memory' OR 'memory, implicit' OR 'repetition priming' OR 'priming, repetition' OR 'bias, implicit' OR 'bias, hidden' OR 'bias, subconscious' OR 'bias, unconscious' OR 'hidden bias' OR 'implicit bias' OR 'subconscious bias' OR 'unconscious bias' OR 'implicit association test') AND ('voice quality' OR 'qualities, voice' OR 'quality, voice' OR 'voice qualities' OR 'speech-language pathology' OR 'language pathology' OR 'pathology, language' OR 'pathology, speech' OR 'pathology, speech language' OR 'pathology, speech-language' OR 'speech and hearing science' OR 'speech and hearing studies' OR 'speech and hearing study' OR 'speech and language pathology and audiology' OR 'audiology' OR 'speech language pathology' OR 'speech pathology' OR 'speech, language and hearing pathology' OR 'speech, language and hearing sciences' OR 'speech, language and hearing studies' OR 'speech, language and hearing study' OR 'speech-language pathology and audiology' OR 'speech-language-hearing pathology') AND [embase]/lim NOT ([embase]/lim AND [medline]/lim)
Web of Science	ALL=(memory OR memories OR "implicit memory" OR "memory, implicit" OR "Repetition Priming" OR "Priming, Repetition" OR "Bias, Implicit" OR "Bias, Hidden" OR "Bias, Subconscious" OR "Bias, Unconscious" OR "Hidden Bias" OR "Implicit Bias" OR "Subconscious Bias" OR "Unconscious Bias" OR "Implicit association test" ) AND ALL=("Voice Quality" OR "Qualities, Voice" OR "Quality, Voice" OR "Voice Qualities" OR "Speech-Language Pathology" OR "Language Pathology" OR "Pathology, Language" OR "Pathology, Speech" OR "Pathology, Speech Language" OR "Pathology, Speech-Language" OR "Speech and Hearing Science" OR "Speech and Hearing Studies" OR "Speech and Hearing Study" OR "Speech and Language Pathology and Audiology" OR "Audiology" OR "Speech Language Pathology" OR "Speech Pathology" OR "Speech, Language and Hearing Pathology" OR "Speech, Language and Hearing Sciences" OR "Speech, Language and Hearing Studies" OR "Speech, Language and Hearing Study" OR "Speech-Language Pathology and Audiology" OR "Speech-Language-Hearing Pathology" )
SCOPUS	( TITLE-ABS-KEY ( memory OR memories OR "implicit memory" OR "memory, implicit" OR "Repetition Priming" OR "Priming, Repetition" OR "Bias, Implicit" OR "Bias, Hidden" OR "Bias, Subconscious" OR "Bias, Unconscious" OR "Hidden Bias" OR "Implicit Bias" OR "Subconscious Bias" OR "Unconscious Bias" OR "Implicit association test" ) AND TITLE-ABS-KEY ( "Voice Quality" OR "Qualities, Voice" OR "Quality, Voice" OR "Voice Qualities" OR "Speech-Language Pathology" OR "Language Pathology" OR "Pathology, Language" OR "Pathology, Speech" OR "Pathology, Speech Language" OR "Pathology, Speech-Language" OR "Speech and Hearing Science" OR "Speech and Hearing Studies" OR "Speech and Hearing Study" OR "Speech and Language Pathology and Audiology" OR "Audiology" OR "Speech Language Pathology" OR "Speech Pathology" OR "Speech, Language and Hearing Pathology" OR "Speech, Language and Hearing Sciences" OR "Speech, Language and Hearing Studies" OR "Speech, Language and Hearing Study" OR "Speech-Language Pathology and Audiology" OR "Speech-Language-Hearing Pathology" ) )
CINAHL	(memory OR memories OR "implicit memory" OR "memory, implicit" OR "Repetition Priming" OR "Priming, Repetition" OR "Bias, Implicit" OR "Bias, Hidden" OR "Bias, Subconscious" OR "Bias, Unconscious" OR "Hidden Bias" OR "Implicit Bias" OR "Subconscious Bias" OR "Unconscious Bias" OR "Implicit association test") AND ("Voice Quality" OR "Qualities, Voice" OR "Quality, Voice" OR "Voice Qualities" OR "Speech-Language Pathology" OR "Language Pathology" OR "Pathology, Language" OR "Pathology, Speech" OR "Pathology, Speech Language" OR "Pathology, Speech-Language" OR "Speech and Hearing Science" OR "Speech and Hearing Studies" OR "Speech and Hearing Study" OR "Speech and Language Pathology and Audiology" OR "Audiology" OR "Speech Language Pathology" OR "Speech Pathology" OR "Speech, Language and Hearing Pathology" OR "Speech, Language and Hearing Sciences" OR "Speech, Language and Hearing Studies" OR "Speech, Language and Hearing Study" OR "Speech-Language Pathology and Audiology" OR "Speech-Language-Hearing Pathology")

Source: The authors

### Identification and selection of relevant studies and data extraction

The data were exported and categorized on the Rayyan Qatar Computing Research Institute (QCRI) platform^(14)^. Two independent researchers performed the first reading, and a third reviewer was invited to assist in decision-making (inclusion or exclusion) in cases of disagreement. Thus, the studies selected at this stage were submitted to full-text reading to analyze their relevance for inclusion in the review.

Texts that addressed implicit memory directly or indirectly, whether in their descriptors, titles, abstracts, objectives, methods, and/or results, were eligible for inclusion. This strategy allowed for an in-depth conceptual evaluation of the content of the articles, ensuring the inclusion of evidence relevant to the phenomenon investigated, even in the absence of standardized terminology in the controlled vocabularies or in the descriptors of the studies.

Data were extracted using a standardized form developed by the authors, including the article title, authors, year and country of publication, type of study, areas, and prevalent themes.

### Assessment of the level of evidence of the studies

The selected articles were classified according to their respective levels of evidence, based on the criteria established by the Oxford Centre for Evidence-Based Medicine^(15)^.

## RESULTS

Initially, 2,565 documents were identified, of which 621 were excluded due to duplication. The remaining 1,944 records were subjected to title and abstract reading, resulting in the exclusion of 1,895 documents. Of the 49 documents selected for full-text reading, 19 were excluded for not meeting the eligibility criteria, and five were excluded for not being available. Six studies that were not initially available in full were obtained by direct request to the authors via email.

At the end of this stage, 25 studies were considered eligible for inclusion. Then, the reviewers conducted a complementary search by analyzing the reference lists of these articles, identifying 21 potentially relevant documents. Five of these were excluded due to duplication, and two due to unavailability of access, resulting in the inclusion of 14 additional studies. Thus, 39^(16-54)^ studies comprised this scoping review, as illustrated in Figure 1.

### Characterization of the studies

The articles were published between 2003 and 2024 in nine countries, with 28^(16-20,22,24,26,28,29,31,32,34,36-41,45-50,52-54)^ from authors in the United States of America, four^(21,25,27,43)^ from Sweden, one^(33)^ from Australia, one^(23)^ from Belgium, one^(42)^ from Brazil, one^(51)^ from Canada, one^(44)^ from Finland, one^(35)^ from the United Kingdom, and one^(30)^ from Taiwan. Table 3 presents the characterization of the studies by themes and methodology.

**Table 3:** Characterization of the studies

**Article**	**Authors/year**	**Country**	**Study objective**	**Population**	**Study type**	**Main results**	**Relevance for the review**
A1^(16)^	Hopper, 2003	USA	To discuss implicit learning in Alzheimer's disease, followed by a summary of the reality of implementing therapeutic programs in long-term care facilities.	Individuals with Alzheimer's disease in long-term care settings	Theoretical/ Review	Implicit learning may be preserved in individuals with dementia, even with impairments in explicit memory. Techniques such as repeated exposure and errorless learning show good results. The speech-language-hearing pathologist should act early and preventively, with a functional focus, including screenings and continuous reassessments. Intervention also involves guidance for caregivers and adaptation of strategies as the disease progresses.	Adult language
A2^(17)^	Anderson and Conture, 2004	USA	To use an age-appropriate version of the syntactic structure priming paradigm to experimentally assess the syntactic processing abilities of children who stutter and children who do not stutter.	Children who stutter and children who do not stutter	Experimental	The main results indicated that children who stutter exhibited slower SRTs in the absence of priming phrases and greater syntactic priming effects than children who do not stutter.	Fluency disorders
A3^(18)^	Turkstra and Bourgeois, 2005	USA	To describe a memory therapy technique, Spaced Retrieval (SR), designed to capitalize on preserved procedural memory processes.	An individual who suffered a traumatic brain injury with profound anterograde amnesia and received SR therapy.	Case study	The positive results support future studies on TBI and suggest considering the role of errors in speech and language therapy.	Adult language
A4^(19)^	Tomblin et al., 2007	USA	To test the hypothesis of deficits in procedural learning by examining serial reaction time learning in adolescents with and without specific language impairment (SLI).	Adolescents with and without SLI	Experimental	Both groups improved on the implicit learning task (SRT), but adolescents with SLI showed slower learning rates. This difference was observed when the impairment was defined by grammar, but not by vocabulary.	Child language
A5^(20)^	Davis et al., 2008	USA	To introduce a novel approach to the treatment of apraxia of speech (AOS) with aphasia using implicit phoneme manipulation via rhyme, omission, and alliteration tasks.	Subject with AOS and mild aphasia.	Experimental	The study showed significant improvement in speech production in children, with small to medium effects for trained sounds (/ʃ/, /ʤ/, /s/). There was generalization to untrained words (T-MAC: 68% to 87%) and noticeable improvement in prosody and articulatory accuracy, confirmed by clinical evaluation by speech-language-hearing pathologists.	Adult language
A6^(21)^	Hedenius et al., 2011	Sweden	To examine the consolidation and long-term learning of procedural sequences in children with SLI.	Children with SLI and typically developing (TD) children	Experimental	Although both groups showed evidence of early sequential learning, only TD children had clear signs of consolidation, although the two groups did not differ in long-term learning. When the children were recategorized based on grammatical deficits rather than broader linguistic deficits, a clearer pattern emerged. While both the group with grammatical impairment and the group with normal grammar showed evidence of early sequential learning, only those with normal grammar showed consolidation and long-term learning. In fact, the group with grammatical impairment appeared to lose any sequential knowledge acquired during the initial testing session. These findings held even when controlling for vocabulary or a broad measure of non-grammatical language, neither of which was associated with procedural memory. When grammar was examined as a continuous variable across all children, the same relationships between procedural memory and grammar were observed, but not vocabulary or the broader measure of language.	Child language
A7^(22)^	Leonard, 2011	USA	To present a tutorial on structural priming and its relevance to the study of grammatical development and language intervention.	Not applicable	Theoretical/ Review	Evidence from the literature on structural priming provides insights into the transition from early conservative grammatical usage to broader abstract grammatical usage in young children and suggests ways in which language intervention activities can be modified to promote greater grammatical changes in children with language difficulties.	Child language
A8^(23)^	Gabriel et al., 2012	Belgium	To evaluate the Procedural Deficit Hypothesis in children with Specific Language Impairment (SLI) using the Serial Reaction Time (SRT) paradigm, establishing an experimental situation characterized by more learning attempts than in previous studies and using a learning sequence that, more specifically, would test the children's ability to learn complex (i.e., second-order) statistical regularities within the sequence.	Fifteen children with SLI and their typically developing (TD) peers	Experimental	Although in Experiment 1 the children with SLI demonstrated learning, they were slower and made more errors than their TD peers. However, these relative weaknesses disappeared when the nature of the response mode changed (Experiment 2).	Child language
A9^(24)^	Silkes et al., 2012	USA	This Phase I study was conducted to investigate the potential effectiveness of masked repetition priming as a treatment for anomia.	Subject with moderate to severe non-fluent aphasia	Experimental	The results revealed a small training effect on the naming of trained items in one category and a pattern of improvement in the other category that did not fully meet the criterion for a small effect. There was also a moderate generalization effect across categories, although no generalization effect was observed for untrained items within semantic categories. Improvements were observed in some measures of general language function.	Adult language
A10^(25)^	Rönnberg et al., 2013	Sweden	To examine the Ease of Language Understanding (ELU) model in light of new behavioral and neural findings on the role of working memory capacity (WMC) in unimodal and bimodal language processing.	Not applicable	Theoretical/ Review	The new ELU model is a meaning prediction system that relies on phonological and semantic interactions in fast implicit and slower explicit processing mechanisms, which depend on WMC, albeit in different ways. It is based on findings that address the relationship between WMC and (a) early attentional processes in speech listening, (b) signal processing in hearing aids and their effects on short-term memory, (c) inhibition of speech maskers and their effect on long-term episodic memory, (d) the effects of hearing impairment on long-term episodic and semantic memory, and finally, (e) auditory effort.	Audiology
A11^(26)^	Valitchka and Turkstra, 2013	USA	To describe the communication patterns between hospitalized patients with declarative memory impairments and rehabilitation team members during the initial stage after injury.	Five adults with acquired brain injury and impairment of declarative memory.	Cross-sectional study	The results showed that staff and visitors frequently asked participants declarative questions whose answers were not verifiable (e.g., questions about events prior to the injury). The verifiable answers were often incorrect but were accepted by staff as correct. The results suggest that acute rehabilitation staff may need training to communicate with patients with declarative memory deficits.	Adult language
A12^(27)^	Hedenius et al., 2013	Sweden	To examine the learning of procedural sequences in DD beyond a single practice session.	Twelve children with developmental dyslexia and 17 controls (typically developing children).	Experimental	The results suggest that the most pronounced impairment in procedural learning in DD may only emerge after prolonged practice, in learning stages beyond a single practice session.	Child language
A13^(28)^	Farias et al., 2014	USA	To promote the understanding and validity of a new treatment for apraxia of speech: implicit phoneme manipulation.	An individual with apraxia of speech	Experimental	Behavioral: The response to implicit training of complex consonant combinations resulted in improvements that were maintained 6 weeks after treatment. Furthermore, this treatment was generalized to simpler consonant combinations in words. Imaging: Functional imaging during implicit phoneme manipulation showed significant activation in brain regions responsible for phonological processing, compared to the baseline semantic task.	Adult language
A14^(29)^	Moring et al., 2014	USA	To investigate whether current measures for tinnitus are susceptible to increased error due to priming and, if so, to examine the feasibility of using the Implicit Association Test for an alternative measurement of tinnitus-related distress.	People with tinnitus	Experimental	Although participants with tinnitus did not consider sound-related words to be significantly more negative, and IAT scores did not predict scores on the Tinnitus Handicap Inventory, priming did affect negative implicit attitudes toward sound-related words.	Audiology
A15^(30)^	Hsu and Bishop, 2014	Taiwan	To test the hypothesis of procedural deficit in specific language impairment (SLI) by comparing the performance of children on two motor procedural learning tasks and one implicit verbal sequence learning task.	Children aged 7 to 11 years with SLI (n = 48), typically developing children with corresponding age (n = 20), and younger typically developing children with corresponding receptive grammar (n = 28).	Experimental	In a serial reaction time task, children with SLI performed at the same level as children with matching grammar but were worse than age-matched controls in learning motor sequences. When tested with a motor procedural learning task that did not involve learning sequential relationships between discrete elements (e.g., chase rotor), children with SLI performed comparably to age-matched children and better than younger grammar-matched controls. Furthermore, poor implicit learning of word sequences was found in a verbal memory task (Hebb effect) in children with SLI.	Child language
A16^(31)^	Silkes, 2015	USA	To investigate the effect of repeated exposure to masked identity stimuli combined with images over multiple trials, sessions, and days on the ability of people with anomia to name these images.	Four participants with anomia	Experimental	All participants showed some progress in identifying the trained items, although to varying degrees, and the trained (prepared) items generally showed greater improvement than the untrained items seen the same number of times. Generalization between categories was observed in some participants, but little or no generalization within the category occurred. Minimal changes occurred in measures of overall language ability.	Adult language
A17^(32)^	Lee and Man, 2017	USA	To examine the feasibility and effectiveness of implicit structural preparation treatment in an individual with agrammatic aphasia.	An individual with aphasia	Case study	MJ significantly improved the production of prepositional dative sentences, both trained and untrained, throughout the training sessions. These effects were maintained after 4 weeks of follow-up without intervention. He also improved notably in the syntactic complexity of connected speech production, such as increased production of verbs and lexical phrases.	Adult language
A17^(33)^	Calder et al., 2017	Australia	To investigate the effectiveness of combined explicit and implicit intervention techniques applied by a speech-language-hearing pathologist to improve receptive and expressive grammar.	Three children (two boys, one girl) aged 6.2–7.0, with grammatical difficulties.	Experimental	Two of the three participants achieved statistically significant gains on standardized tests of receptive and expressive general grammar. Two of the three children showed statistically significant improvement in measures of expressive morphosyntax, with one participant continuing to improve 5 weeks after treatment.	Child language
A19^(34)^	Walden and Lesner, 2018	USA	This study assessed implicit and explicit attitudes toward people who stutter among young adults with normal fluency.	Adults without stuttering, or with typical fluency, moving towards stuttered speech.	Cross-sectional study	The results corroborated the existence of a negative stereotype of stuttering. Participants demonstrated implicit and explicit negative attitudes toward people who stutter. Explicit attitudes toward stutterers, but not implicit attitudes, were significantly predicted by social desirability scores. Familiarity with stuttering was significantly associated with implicit, but not explicit, attitudes toward stuttering.	Fluency disorders
A20^(35)^	Garraffa et al., 2018	United Kingdom	To investigate whether a group of children with Developmental Learning Disorder showed impairment in implicit syntax learning (syntactic priming) after individual syntactic experiences, and the temporal course of such effects.	Children aged 5 to 6 years, Italian speakers, with Developmental Learning Disorder and typically developing controls, matched for age and language.	Experimental	Children with Developmental Learning Disorder showed immediate syntactic priming, but it was less intense and less lasting than that of typically developing children of the same age. The effect did not persist after intermediate sentences, and there was no cumulative priming effect in any group.	Child language
A21^(36)^	Lee et al., 2019	USA	To examine whether people with aphasia exhibit preserved and lasting structural priming effects during the interpretation of syntactically ambiguous sentences, and whether these priming effects occur independently or in conjunction with lexical (verbal) information.	People with aphasia and healthy older adults	Experimental	After reading a priming sentence with a specific interpretation, healthy older adults and people with aphasia tended to interpret an ambiguous priming sentence in a target sentence in the same way and with faster response times. Importantly, both groups continued to exhibit this priming effect over time (Experiment 2), although the effect was not as reliable in terms of response times. However, neither group showed lexical (verb-specific) priming enhancement, deviating from the strong lexical enhancement observed in young adults in previous studies.	Adult language
A22^(37)^	Man et al., 2019	USA	To investigate whether lexically independent priming (abstract structure) and/or lexically specific priming (verb) produces an immediate and more lasting facilitation of syntactic production in people with aphasia.	Seventeen individuals with aphasia (PWA) and 20 healthy older individuals	Experimental	Healthy older adults demonstrated abstract priming in transitive and dative cases, not only in the immediate priming condition (Experiment 1) but also in the long-lasting priming condition (Experiment 2). They also demonstrated significantly increased priming by verbal overlap (lexical reinforcement) in transitive cases during immediate priming. Individuals with aphasia demonstrated abstract priming in transitive cases in both immediate and long-lasting priming conditions. However, the magnitude of priming was not increased by verbal overlap.	Adult language
A23^(38)^	Kamhi, 2019	USA	To analyze how the development of speech and language can be viewed as proceduralization and skill learning.	Individuals with speech and language disorders.	Theoretical/ Review	Norm-referenced measures provide a good measure of deliberative speech and language processes, while social conversations and discourses on familiar topics with familiar listeners provide the best indication of proceduralized speech and language processes. Intervention goals and planning need to consider which speech and language operations should be proceduralized and which will primarily require deliberative processing. Further research and clinical application of these ideas are clearly needed to develop more precise criteria that define the dividing line between deliberative and proceduralized speech and language processes.	Child language
A24^(39)^	Walden et al., 2020	USA	To assess implicit and explicit attitudes toward stuttering and those who stutter among speech-language-hearing pathologists.	Speech-language-hearing pathologists	Cross-sectional study	As a group, clinicians demonstrated negative implicit attitudes toward stuttering. Explicit attitudes toward a person who stutters were positive, although less positive than attitudes toward a person who does not stutter. The amount of prior exposure to stuttering among these experienced speech-language-hearing pathologists was not significantly associated with implicit or explicit attitudes.	Fluency disorders
A25^(40)^	Wada et al., 2020	USA	To examine the use of structural priming combined with a focused reformulation procedure to obtain subject- and object-centered relative clauses, in students with developmental language disorders (DLD) and typically developing (TD) students.	26 children (13 DLD, 13 TD), aged 6;10 to 10;11 (years; months)	Experimental	DLD and TD children were more accurate in producing subject-focused relative clauses than object-focused ones. However, children with DLD required more attempts to achieve consistent performance in both types of structures, indicating a slower learning rate.	Child language
A26^(41)^	Evans, 2021	USA	To discuss the concept of implicit bias in the field of audiology.	Not applicable	Theoretical/ Review	This article discusses implicit biases and prejudices in audiological practice, including the underrepresentation of Black professionals, socioeconomic and linguistic bias, and audism. It highlights how these factors affect the way information is shared with families and proposes an affirmative and culturally sensitive approach to clinical communication.	Audiology
A27^(42)^	Maziero et al., 2021	Brazil	To compare the performance of individuals with mild cognitive impairment (MCI) with that of healthy older adults in a text inference comprehension task, and to determine the best predictors of performance in this skill, considering a verbal episodic memory task and two semantic tasks.	Individuals with mild cognitive impairment and healthy older adults	Cross-sectional study	Patients with MCI performed worse than healthy older adults; there were no differences between the MCI subgroups (amnesic and non-amnesic). The best predictors for making inferences were verbal memory in the MCI group and semantic tasks in the non-MCI group.	Adult language
A28^(43)^	Hedenius et al., 2021	Sweden	To examine whether prolonged initial practice in Session 1 would normalize procedural consolidation in children with developmental dyslexia.	Children with Developmental Dyslexia and typically developing children	Experimental	The amount of sequence learning was similar in both groups at the end of session 1 (p=0.797, η ρ 2 = 0.001). In the follow-up session after 24 hours, the DD group performed worse than the TD group (p=0.003, η ρ 2 = 0.129). The amount of sequence knowledge in Session 2 predicted a single variance in reading fluency (p=0.024, η ρ 2 = 0.083), independently of the children's phonemic awareness and inattention symptoms.	Child language
A29^(44)^	Kautto and Mainela-Arnold, 2022	Finland	Deficits in procedural learning have been hypothesized to contribute to language, especially grammatical difficulties, in individuals with specific language impairment (SLI). This study tested this hypothesis by examining serial reaction time (SRT) learning in adolescents with and without SLI.	Adolescents who had been identified with SLI and adolescents with normal language.	Experimental	Adolescents with SLI showed slower learning rates during pattern learning than controls. When language impairment was defined in terms of grammatical impairments, similar slower learning rates were found, but when language impairment was based on vocabulary, no group differences were found.	Child language
A30^(45)^	Baron and Arbel, 2022	USA	To present evidence of the interaction between implicit and explicit learning systems and to highlight the characteristics of the intervention that promote implicit or explicit learning, as well as outcome measures that explore implicit or explicit knowledge.	Not applicable	Theoretical/ Review	Many intervention features (e.g., instructions, elicitation techniques, feedback) can be manipulated to move an intervention along the implicit-explicit continuum. Given the bias toward the use of explicit learning strategies that develops throughout childhood and into adulthood, clinicians should be aware that most interventions (even those that promote implicit learning) will engage the explicit learning system. However, a greater awareness of both implicit and explicit learning systems and their cognitive demands will allow clinicians to choose the most appropriate intervention for the target behavior.	Child language
A31^(46)^	Tabrizi et al., 2022	USA	Repetitive priming has been suggested as a method for addressing implicit processes in the treatment of anomia. Previous studies have used masked priming for this purpose. This study expands on that work with visible priming, a more clinically feasible approach.	Three participants with aphasia.	Experimental	All participants improved in identifying trained items immediately after treatment, with greater improvements for trained items than for untrained items. All participants maintained some degree of improvement on trained items 3 months after treatment, although the degree varied among participants. Inconsistent generalization occurred for unexposed items. Improvements were observed in some areas of broader language, although these varied.	Adult language
A32^(47)^	Norotsky et al., 2023	USA	To understand the role of implicit racial bias in auditory-perceptual evaluations of dysphonic voices, determining whether there is a bias effect for novice listeners in their auditory-perceptual evaluations of Black and White speakers.	Postgraduate students in speech and language pathology at Boston University	Experimental	Both Black and White speakers were classified as less severely dysphonic when their race was labeled as Black.	Voice
A33^(48)^	Lopez et al., 2023	USA	To determine how the manner of L2 acquisition can affect interlinguistic generalization after L2 treatment, focusing separately on lexicon (object naming) and grammar (sentence construction).	Two Spanish-English bilinguals with aphasia.	Experimental	As predicted, P2 showed interlanguage generalization after verb/grammar treatment (VNeST), while P1 did not. However, contrary to the prediction that both participants should show interlanguage generalization after noun treatment (SFA), only P1 showed interlanguage generalization in object naming.	Adult language
A34^(49)^	Silkes, 2023	USA	To expand the evidence for the treatment of anomia based on repetition priming.	Five participants with chronic aphasia	Quasi-experimental	Items previously activated by priming resulted in greater improvements in naming than those that were only practiced but without priming. The data suggest that the gains may extend beyond the direct association with the trained stimuli. There was variation in the maintenance and generalization effects.	Adult language
A35^(50)^	Lee et al., 2024	USA	To examine the impact of aging on the strength and longevity of the effects of abstract structural priming and lexical reinforcement.	Younger and older adults	Experimental	In immediate priming, older adults showed abstract structural priming and lexical increase in transitive and dative cases, similar to that observed in younger adults. In long-term priming, only the effects of abstract priming were significant in both groups of participants, with no evidence of age-related priming reduction.	Adult language
A36^(51)^	Beadle et al., 2024	Canada	To investigate the perceptions of older and younger adults about older and younger adults who use hearing aids.	Thirty older adults and 30 younger adults who reported not having hearing aids or diagnosed hearing impairment.	Cross-sectional study	In both IATs, older and younger participants responded faster and more accurately when images of individuals using hearing aids were paired with negative words compared to positive words. Photo ratings did not vary in relation to the presence or absence of hearing aids for any age group.	Audiology
A37^(52)^	Montgomery et al., 2024	USA	We propose that implicit learning, including syntactic preparation, holds therapeutic promise for improving the syntactic knowledge of children with developmental language disorder (DLD).	Children with developmental language disorders (DLD).	Theoretical/ Review	Statistically induced block-based learning is a powerful driver of syntactic learning, and syntactic priming is one form of this learning. Repeated episodes of priming during everyday linguistic interactions lead children to create abstract and global syntactic representations in long-term memory. We offer some reflections on an implicit language intervention approach focusing on syntactic priming.	Child language
A38^(53)^	Balthazar and Scott, 2024	USA	To present key concepts that highlight the importance of guiding schoolchildren and adolescents with developmental language disorders (DLD) through complex sentences.	Schoolchildren and adolescents with DLD	Theoretical/ Review	Complex sentence structures represent a fundamental challenge for students with DLD when dealing with discipline-specific academic and linguistic tasks. Sentence complexity treatment programs employ one or more treatment methods, including priming, modeling, reformulation, contextualization, metalinguistic instruction, and sentence combination. Although studies have consistently demonstrated measurable improvement in complex sentence production in proximal outcomes, regardless of the treatment approach, evidence of lasting functional changes in students with DLD remains scarce. We encourage new treatments that target the comprehension and production of complex sentences in real-life academic contexts, clinical practice, and research.	Child language
A39^(54)^	Morton-Jones and Sund, 2024	USA	To explain the impactful role that speech-language-hearing pathologists and voice clinicians can play in daily clinical practice to promote health equity, mitigating the well-documented negative effects of implicit personal biases in the health field and offering culturally responsive practices.	Speech-language-hearing pathologists and voice clinicians	Theoretical/ Review	Evidence suggests that implicit bias has a serious impact on daily clinical practice and dialogue. Within the complexities of accessing healthcare, a clinician creates barriers or enablers that determine the acceptability and accessibility of the care offered. Unaddressed implicit bias and a lack of culturally responsive practices can reduce the quality of care and ultimately prevent a patient from seeking behavioral voice services.	Voice

Source: The authors.

Of the included studies, 22^(17,19-21,23,24,27-31,33,35-37,40,43,44,46-48,50)^ were experimental, nine^(16,22,25,38,41,45,52-54)^ were theoretical studies, five^(26,34,39,42,51)^ were cross-sectional observational studies, two^(18,32)^ were case studies, and one^(49)^ was a quasi-experimental study.

The most prevalent areas were related to language, totaling 33^(16-24,26-28,30-40,42-46,48-50,52,53)^ articles (84.6%), distributed among adult language (15 articles)^(16,18,20,24,26,28,31,32,36,37,42,46,48-50)^, child language (15 articles) ^(19,21-23,27,30,33,35,38,40,43-45,52,53)^ and fluency disorders and impairments (three articles)^(17,34,39)^. The remaining studies addressed topics related to voice – two articles^(47,54)^ (5.1%) – and audiology – four articles^(25,29,41,51)^ (10.3%).

**Figure 2:**
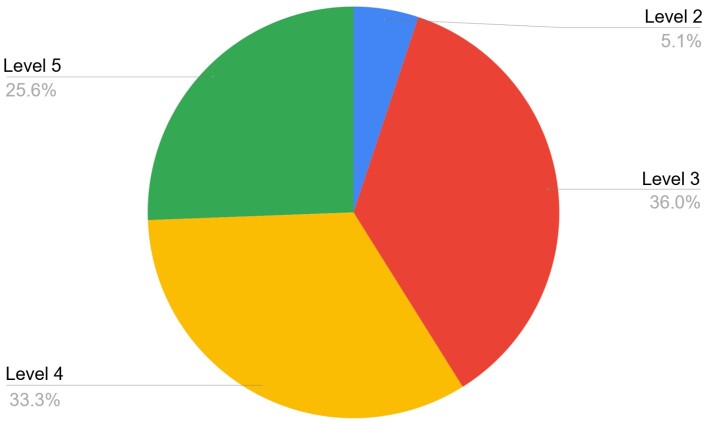
Breakdown of included articles based on the levels of evidence of the Oxford Centre for Evidence-Based Medicine

Source: Based on OCEBM^(15)^

Figure 2 shows the characterization of the studies regarding their level of scientific evidence^(15)^. Level 1 represents the highest, and Level 5 the lowest level of evidence.

## DISCUSSION

In general, the texts address the applicability of implicit memory in SLH practice. Given the diversity of findings, it was decided to organize the discussion into five topics, based on the SLH areas most frequently addressed in the studies: 1. language (1.1. adult language, 1.2. child language, and 1.3. fluency disorders and impairments) 2. audiology; 3. voice.

### Language

#### Adult language

Themes related to language in adults were recurrent in 15^(16,18,20,24,26,28,31,32,36,37,42,46,48-50)^ studies, which shows a high prevalence of studies that addressed implicit memory in this population. The studies focused on the application of implicit learning in SLH therapy and the use of implicit tasks for the assessment and characterization of disorders such as aphasia and apraxia of speech, mild cognitive impairment, dementia/Alzheimer's disease, declarative memory impairment, and traumatic brain injury with deep anterograde amnesia.

The study by Lee et al.^(36)^ investigated whether people with aphasia have preserved and lasting structural priming effects during the interpretation of syntactically ambiguous sentences, and whether such effects occur independently or in conjunction with lexical (verbal) information. Structural priming refers to an individual's involuntary tendency to repeat a previously heard or read syntactic structure^(55)^. The results indicated that people with aphasia continue to benefit from abstract structural priming (i.e., repeated exposure to grammatical structures), even in the absence of repetition of specific lexical items. This mechanism contributes to language comprehension and retention, proving to be functional both immediately and in the long term.

These findings are consistent with the study by Lee and Man^(32)^, which investigated the effectiveness of structural priming as an intervention in an individual with agrammatic aphasia. The results suggest that the use of implicit structural priming may be a viable approach to the rehabilitation of syntactic production in aphasia, promoting overall long-term language recovery by strengthening connections between linguistic representations, based on experience.

Regarding structural priming, Man et al.^(37)^ investigated whether abstract structural priming and/or lexically specific priming (with the same verb) promote immediate and lasting facilitation of syntactic production in people with aphasia. The results showed that participants with aphasia exhibited effects of abstract structural priming in transitive sentences, similarly to healthy older adults, in both immediate and sustained measures. However, unlike the control group, the group with aphasia did not benefit from lexical reinforcement, suggesting a limitation in the use of lexical cues to support syntactic production. Lee et al.^(50)^ observed that both lexically specific and independent mechanisms of structural priming remain preserved in older adults, which corroborates the idea that structural priming reflects a lifelong linguistic learning mechanism.

López et al.^(48)^ also investigated aphasia in the context of language treatment for aphasic bilinguals, aiming to understand how the mode of second language (L2) acquisition (explicit or implicit) can influence interlinguistic generalization after L2 intervention. The study distinguished the effects of treatment at the lexical level (object naming) and at the grammatical level (sentence construction), analyzing two bilingual participants with aphasia: P1, who learned L2 explicitly, and P2, who acquired L2 implicitly. Interlinguistic generalization was observed in both cases, but in different language components. The results suggest that naturalistic (implicit) L2 learning tends to strengthen grammatical links between languages, while formal (explicit) learning promotes more robust connections in the lexical-semantic system.

Another strategy used in the treatment of aphasia is masked repetition priming, used by Silkes et al.^(24)^ to intervene in anomia. Masked visual priming involves the visual presentation of a main item preceded and/or followed by other visual stimuli that serve to interrupt the brain's conscious recognition of the main item. Based on this concept, the purpose of this Phase I study, with a single participant and multiple baselines, was to investigate whether repeated training with masked repetition priming has cumulative positive effects on lexical retrieval, which would support its potential as an alternative or complementary treatment for anomia. Thus, the networks would be strengthened and develop automatically and unconsciously, with limited or no influence from conscious processing. The results suggested that masked visual priming can change naming ability over time and may have the potential to improve word retrieval in individuals with anomia. These results corroborate the Phase II study by the same authors^(31)^.

Meanwhile, Tabrizi et al.^(46)^ addressed visible repetition priming in the treatment of anomia with the justification that it is more clinically feasible. Visible priming in this study demonstrated that this treatment could increase naming accuracy for individuals with anomia and benefit other aspects of language. This study agrees with the study by Silkes^(49)^, in which the application of repetition priming in five individuals with aphasia demonstrated that items previously activated by priming resulted in greater improvements in naming than those only practiced but without priming. The data suggest that the gains may go beyond direct association with the trained stimuli.

Implicit phoneme manipulation has been used as a therapeutic strategy in apraxia of speech. Experimental studies with a single-subject, multiple baseline design, conducted by Davis et al.^(20)^ and Farias et al.^(28)^, have demonstrated positive results with this approach. Participants improved in spontaneous speech production and accuracy of trained words, including reduced sound distortions, decreased phonological errors, and improved prosody. Both studies also reported significant generalization to untreated words. In addition, neuroimaging evidence indicated that this approach activates brain areas involved in phonological processing, planning, and motor programming of speech.

Hopper's study^(16)^ discusses implicit learning in individuals with Alzheimer's disease, showing that this type of learning can be relatively preserved even in the presence of significant impairments in explicit memory. This finding was based on the observation that participants with dementia were able to learn functional information through techniques such as spaced retrieval or repeated exposure, even without declarative awareness of learning. These findings provide a solid rationale for SLH interventions that prioritize functional communication. In this sense, early and preventive action by the SLH pathologist is essential, focusing on strategies such as periodic screenings, continuous reassessments, and methods such as errorless learning, which avoids reinforcing incorrect responses and has proven effective in this group. Thus, the aim is to take advantage of preserved procedural memory and minimize the demands of declarative learning^(16,18)^.

Although it does not focus on implicit memory, the study by Maziero et al.^(42)^ contributes to this field of knowledge by using the Implicit Management Test to investigate the performance of older adults with mild cognitive impairment. This test is a measure that assesses the ability to understand inferences during reading activities through texts that contain explicit and implicit information, necessary for correct interpretation during reading. The findings highlight that, even in the face of deficits in episodic memory and explicit tasks, aspects of implicit processing can still exert an important influence on textual comprehension. This type of evidence reinforces the relevance of considering therapeutic strategies based on alternative cognitive pathways and less dependent on conscious recall in SLH intervention.

#### Child language

Themes related to child language were recurrent in 15^(19,21-23,27,30,33,35,38,40,43-45,52,53)^ studies.

Within the field of child language, one of the most frequently addressed themes was the procedural deficit hypothesis (PDH) in developmental language disorder (DLD)^(19,21,23,30)^. This theory was proposed by Ullman and Pierpont^(56)^ to explain the linguistic and non-linguistic problems observed in children with DLD. The PDH is based on the declarative/procedural model of language learning, according to which lexical acquisitions are closely associated with declarative memory, while procedural learning supports various aspects of grammar. From this perspective, declarative memory processes the linking of conceptual, phonological, and semantic representations, while procedural memory, which is one of several systems involved in implicit acquisition, supports aspects of rule learning and is particularly important for the acquisition and use of skills involving sequences, whether abstract, sensorimotor, or cognitive^(57)^.

It should be noted that some studies^(19,21,23,30)^ use the nomenclature “specific language impairment” (SLI) or “specific language disorder” (SLD), currently replaced by DLD, according to the recommendations of the CATALISE project^(58,59)^.

Tomblin et al.^(19)^ compared adolescents with SLI and typical development (TD) using the serial reaction time (SRT) paradigm, an implicit sequence learning task that measures the time it takes the participant to respond correctly (for example, pressing the key in the box where an image appears). They found that both groups improved on the implicit learning task (SRT), but adolescents with SLI had slower learning rates. This difference was observed when the impairment was defined by grammar, but not by vocabulary^(19,21,23,30)^.

Gabriel et al.^(23)^ conducted a similar study, but with two experiments: the first using a keyboard as a response mode and the second using a touchscreen, which would reduce the motor and cognitive demands of the task. In the first experiment, with responses via keyboard, children with SLI were slower and made more mistakes; in the second, with responses on a touchscreen, these differences disappeared, suggesting that the previous difficulties may have been linked to motor demands and not to procedural learning deficits. Therefore, this study contradicts that of Tomblin et al.^(19)^, suggesting that language impairments in DLD are not underpinned by deficient procedural learning skills.

The study by Hsu and Bishop^(30)^ supported this conclusion by using the SRT and a motor procedural learning task that did not involve learning sequential relationships between discrete elements to test children with SLI, typically developing children of corresponding age, and younger typically developing children with corresponding receptive grammar. The results showed that children with SLI had the same level of performance as children with corresponding grammar but were worse than age-matched controls in learning motor sequences. In the same study, children with SLI performed similarly to age-matched children and better than younger controls with corresponding grammar in the motor procedural learning task. From this result, it can be concluded that deficits in SLI are more linked to learning sequential information than to a generalized deficiency in procedural learning.

The study by Hedenius et al.^(21)^ examined the consolidation and long-term learning of procedural sequences in children with SLI, using alternating serial reaction time (ASRT), which differs from SRT because random trials are alternated with trials following the fixed sequence throughout the task. The results indicated impairments in consolidation and long-term learning, especially associated with grammatical deficits, corroborating the idea that language difficulties are related to specific, not generalized, deficits in procedural learning.

In addition to investigating cognitive mechanisms in SLI, some studies focus on therapeutic strategies related to the treatment of this disorder. One of the prominent approaches is structural priming, used both in studies with children with SLI and in research on aphasia. Children with SLI demonstrated a slower learning rate with structural priming in the production of relative clauses focusing on the subject and object, compared to typically developing children^(19)^.

In this context, Wada et al.^(40)^ explored the feasibility of combining implicit priming tasks with explicit reformulation in teaching subject-focused relative clauses, showing that such strategies can be effective, although a greater number of trials or additional instructions are probably needed for children with SLI to achieve consistent performance.

Additionally, Baron and Arbel^(45)^ published a tutorial that discusses how different characteristics of SLH interventions, such as instructions, feedback, and elicitation techniques, influence the involvement of implicit and explicit learning systems. The authors highlight that, due to developmental bias, most interventions tend to predominantly involve the explicit system, both in children and adults. However, understanding both systems allows clinicians to adjust their therapeutic choices to the target behavior, positioning the intervention along a continuum between implicit and explicit. An applied study cited in the tutorial demonstrated significant gains in standardized tests of receptive and expressive general grammar and expressive morphosyntax from the combined use of implicit and explicit techniques by an SLH pathologist.

Syntactic priming has also been used as a strategy to assess implicit syntax learning in children with SLI. The study by Calder et al.^(33)^ showed that, although children with SLD exhibit immediate syntactic priming effects, these tend to be less intense and lasting than in typically developing peers. This suggests that these children have difficulty learning grammatical patterns implicitly, extracting less useful information from each linguistic exposure, which hinders the gradual construction of more complex syntactic knowledge.

In this sense, the theoretical study by Montgomery et al.^(52)^ highlights that learning syntactic structures depends on repeated experiences of syntactic priming. Thus, including activities of this type in the intervention repertoire can strengthen syntactic knowledge and improve sentence comprehension and production in children with grammatical difficulties.

Studies on implicit learning in developmental dyslexia (DD) have also been identified. One of the most established theoretical hypotheses in the field suggests that the deficits observed in DD are associated with a dysfunction in the procedural memory system, especially involving the cortico-cerebellar and/or cortico-striatal circuits^(60)^.

In this context, Hedenius et al.^(21)^ conducted a series of studies in 2011 to deepen the understanding of procedural learning mechanisms in children with DD. In 2013^(27)^, the authors investigated the performance of these children not only during the initial practice session, but also in later stages, including nighttime consolidation and practice on a second day. The results suggested that the most pronounced procedural learning deficits emerge after prolonged practice, indicating that the difficulties associated with DD may manifest more strongly in the later stages of learning, which reinforces the hypothesis of dysfunction in procedural memory.

In a later study, published in 2021, Hedenius et al.^(43)^ tested whether longer initial practice could compensate for consolidation difficulties. Although this prolonged practice was implemented, the deficits in procedural consolidation persisted, suggesting that the learning problems observed earlier are not restricted to the initial phase but are more related to memory consolidation over time, especially during sleep.

#### Fluency disorders and impairments

Three studies on stuttering were identified^(17,34,39)^. Two of them used the Implicit Association Test (IAT)^(34,39)^ to assess implicit attitudes towards individuals who stutter. The IAT is an instrument that assesses response latency in the association between target categories and evaluative attributes, based on the principle that associations more strongly consolidated in implicit memory are processed more quickly and with fewer errors. The IAT, therefore, allows the measurement of implicit attitudes, which may differ from the attitudes explicitly stated by individuals^(61)^. The first study, conducted by Walden and Lesnar^(34)^, aimed to investigate the implicit and explicit attitudes of young adults with typical fluency towards people who stutter. They applied specific scales and a social desirability scale to assess explicit attitudes. The results confirmed the existence of negative stereotypes associated with stuttering, with participants demonstrating both implicit and explicit negative attitudes.

The second study with the IAT, by Walden et al.^(39)^, had a similar focus, but with SLH pathologists as participants. In this case, explicit attitudes toward people who stutter were generally positive, although less favorable than toward people who do not stutter. Implicit attitudes, however, were negative. These findings highlight the importance of considering implicit biases in professional training, since unintentional negative attitudes can impact therapeutic engagement and empathy in the clinician-patient relationship.

The third study, by Anderson and Conture^(17)^, examined syntactic priming in children who stutter (CWS) and typically developing children (CWNS). CWS participants had slower reactions in the absence of priming phrases and demonstrated more intense syntactic priming effects, suggesting a greater reliance on external structural cues. These findings indicate that stuttering may be related to difficulties in accessing syntactic representations quickly and efficiently, which may contribute to disfluency. Such evidence points to the therapeutic potential of strategies that explore the implicit structuring of language.

### Audiology

Four studies in the field of audiology were identified^(25,29,41,51)^, of which two^(29,51)^ used IAT.

The study by Beadle et al.^(51)^ investigated the perceptions of older and younger adults about same-age people who use hearing aids. To do this, participants completed two implicit association tests: one with images of older adults and another with younger adults, both with and without hearing aids. They also assessed the age, attractiveness, and intelligence of the people portrayed. While explicit judgment tasks indicated neutral attitudes towards hearing aid users, the IAT results suggest the presence of negative implicit attitudes, since participants responded more quickly and with greater accuracy when images of hearing aid users were associated with negative words, compared to positive words.

The study by Moring et al.^(29)^ evaluated the feasibility of using IAT to measure attitudes related to tinnitus and investigate whether traditional instruments, such as the Tinnitus Handicap Inventory (THI), would function as priming stimuli. Although the words related to tinnitus did not generate strong implicit associations, and the IAT scores did not predict the THI scores, the authors observed that the prior completion of explicit questionnaires can negatively influence IAT results by activating a negative bias in the responses. Thus, the study suggests that real auditory stimuli (such as tinnitus sounds) may be more effective than written words in activating implicit structures related to tinnitus.

The study by Evans^(41)^ addresses implicit biases and prejudices in audiological practice, including the underrepresentation of Black professionals, socioeconomic and linguistic bias, and audism. It highlights how these factors affect the way information is shared with families and proposes an affirmative and culturally sensitive approach to clinical communication. It suggests reflecting on the postgraduate curriculum and how it can more accurately reflect the communities it serves.

In addition to studies investigating implicit attitudes in audiology, one article was identified that, while not focusing centrally on implicit learning, addresses its role in language processing under adverse auditory conditions. The study by Rönnberg et al.^(25)^ proposes an update to the Ease of Language Understanding (ELU) model, which describes how working memory interacts with implicit and explicit processing mechanisms in speech comprehension. The model suggests that fast implicit processing is essential for automatic speech recognition when the auditory signal is clear, while explicit processing (slower and dependent on working memory) is recruited when there are distortions in the signal, such as in noisy situations or when using hearing aids. Although the main focus is on working memory, recognizing the role of implicit mechanisms in this model broadens the understanding of how auditory processing interacts with different learning systems, especially in populations with hearing loss.

### Voice

The two^(47,55)^ studies found in the area of ​​voice address implicit biases in vocal clinical practice. The first, by Norotsky et al.^(47)^, investigated the role of implicit racial bias in auditory-perceptual evaluation of dysphonic voices, aiming to verify whether the speaker's race influenced the assessments made by novice listeners. SLH postgraduate students listened to recordings of Black and White speakers with vocal disorders, assessed the voice using a 100-unit visual analog scale, and then performed the IAT to measure their racial bias. The results suggested minimizing bias in the assessments of Black speakers, indicating that Black patients with vocal disorders may receive less severe assessments, which may compromise proper diagnosis and treatment.

These findings are further developed in the study by Morton-Jones and Sund^(54)^, which more broadly discusses the impact of implicit bias on clinical voice practice. The article highlights that unconscious bias and the absence of culturally responsive practices can compromise the quality of SLH care and even discourage patients from seeking vocal treatment. Therefore, the authors reinforce the need for SLH pathologists and interdisciplinary teams to develop conscious strategies to address implicit biases, promoting more ethical, equitable, and inclusive care.

#### Regarding the evaluation of methodological quality

The evaluation of the level of evidence of the studies indicated that most studies fall into level 3 (36.0%), indicating a predominance of evidence derived from observational studies, cohort studies, or case-control studies, while only 5.1% reach level 2, corresponding to evidence from well-conducted randomized clinical trials. Levels 4 and 5, which represent case series studies, case reports, or expert opinions, correspond to 3.3% and 25.6% of the studies, respectively. These findings reflect the methodological heterogeneity of the literature on implicit memory in SLH pathology and suggest that, although experimental studies exist, much of the evidence is still based on observational designs or reports, which reinforces the need for investigations with more robust designs and methodological standardization to consolidate the knowledge base and support clinical practices more grounded in evidence.

### Limitations and contributions for the future

As a limitation from a conceptual standpoint, the included studies lack standardized terms and are somewhat ambiguous in the definition of implicit memory. Regarding the assessment instruments, the diversity of tasks used and the lack of standardization may hinder comparisons between the findings. Finally, regarding the generalization of the results, the studies focus mainly on some SLH areas, such as language, audiology, and voice, suggesting the possibility of investigation in other clinical contexts and sub-areas of the field.

One of the contributions of this review is to point out to specialists the existing gaps in other SLH areas as a potential field of investigation with clinical applicability.

## CONCLUSION

International scientific SLH production has addressed implicit memory in a still incipient and heterogeneous way, focusing mainly on the areas of language, audiology, and voice. There is a predominance of studies exploring implicit learning as a therapeutic resource, the use of implicit tasks for the assessment and characterization of disorders, and investigations into biases and implicit perceptions in SLH practice. Despite highlighting the potential of implicit memory to expand assessment and intervention strategies, the literature presents conceptual and methodological limitations, including the absence of terminological standardization and the diversity of designs and instruments used. These findings indicate the need for more systematized future investigations, with greater conceptual and methodological clarity, to consolidate the role of implicit memory in SLH pathology and to support evidence-based clinical practices.
