# Supplementary material for: What has been researched about implicit memory in speech-language-hearing pathology? A scoping review
Source: Codas. 2026 Aug 3;38(5):e20250338. doi: 10.1590/2317-1782/e20250338en (PMC13445993; doi:10.1590/2317-1782/e20250338en)
Supplement: Translation

# Translation: O que se tem pesquisado sobre a memória implícita na fonoaudiologia? Uma revisão de escopo

**DOI:** 10.1590/2317-1782/e20250338pt

**Authors:** Fernanda Roberta de Faria Rocha da Silva, Gilberto da Cruz Leal, Roberta de Carvalho Barabás, Katia Nemr

**Affiliations:** 1 Departamento de Fisioterapia, Fonoaudiologia e Terapia Ocupacional – FOFITO, Faculdade de Medicina, Universidade de São Paulo – USP - São Paulo (SP), Brasil.; 2 Instituto Claretiano de Ensino - Batatais (SP), Brasil.

**Keywords:** Priming de Repetição, Memória Implícita, Fonoaudiologia, Viés Implícito, Revisão de Escopo

## Abstract

**Objetivo:**

mapear como a memória implícita tem sido abordada na produção científica internacional em Fonoaudiologia.

**Estratégia de pesquisa:**

Foi realizada busca eletrônica nas bases PubMed, Web of Science, Scopus, Embase, CINAHL e PsycINFO, complementada por busca manual nas referências dos estudos incluídos. A revisão de escopo foi conduzida com base no Manual para Síntese de Evidências do Joanna Briggs Institute, seguindo as diretrizes PRISMA-ScR. O protocolo foi registrado no Open Science Framework (10.17605/OSF.IO/PNDWF).

**Critérios de seleção:**

Foram incluídos estudos que abordassem a memória implícita em contextos fonoaudiológicos, em português, inglês ou espanhol, independentemente do ano de publicação.

**Análise dos dados:**

Dois revisores extraíram os dados de forma independente, considerando delineamento, população, objetivos e principais desfechos. Os estudos foram agrupados por área de aplicação e sintetizados por análise descritiva e narrativa.

**Resultados:**

Um total de 2.565 documentos foram identificados, dentre os quais 39 compuseram essa revisão. Os estudos foram agrupados em 3 categorias (linguagem, audiologia e voz). Os temas mais frequentes encontrados foram: a aplicação da aprendizagem implícita em contextos terapêuticos fonoaudiológicos; o uso de tarefas implícitas para avaliação e caracterização de transtornos; as investigações de vieses, preconceitos e/ou percepções implícitas na prática fonoaudiológica.

**Conclusão:**

A literatura trata a memória implícita de forma limitada e heterogênea, principalmente em linguagem, audiologia e voz, com foco em aprendizagem implícita, avaliação e vieses. Apesar do potencial clínico, há carência de sistematização conceitual e metodológica, indicando a necessidade de pesquisas futuras para consolidar seu uso na área.

## INTRODUÇÃO

Por volta da década de 1970, o estudo da memória humana passou por uma considerável transformação que, segundo Richardson-Klavehn e Bjork^(1)^, constituiu uma revolução na forma como medimos e interpretamos a influência de eventos passados na experiência e no comportamento^(1)^. O marco central dessa mudança foi o reconhecimento de que a memória de experiências anteriores pode ser expressa por duas vias distintas: a memória explícita e a memória implícita^(2)^.

A memória explícita refere-se à evocação consciente de informações recentemente adquiridas. Em testes que analisam esse tipo de memória, os indivíduos são expostos a materiais - como palavras ou figuras - e, posteriormente, solicitados a lembrar ou reconhecer esses itens conscientemente. A maior parte dos estudos sobre memória humana tem se concentrado nesse tipo de processo^(2,3)^.

Diferentemente da memória explícita, que é consciente, a memória implícita é inconsciente, envolvendo conteúdos que não são evocados por meio de palavras, mas por desempenho^(4)^. A avaliação da memória implícita ocorre por meio de tarefas que não exigem a recordação consciente dos episódios prévios, como por exemplo, completar palavras que são apresentadas de forma fragmentada, preferir um estímulo previamente exposto em meio a um grupo de estímulos e/ou ler textos invertidos^(2)^.

Entre as manifestações da memória implícita, o fenômeno mais estudado é o priming ou repetição, no qual a exposição prévia a uma palavra ou objeto facilita seu reconhecimento ou processamento posterior, diante de pistas perceptivas mínimas^(5)^. Além do priming, diversas formas de aprendizagem perceptiva, motora e cognitiva também são consideradas expressões da memória implícita, desde que ocorram sem referência consciente aos episódios de aprendizagem anteriores. Em termos operacionais, o priming é geralmente observado após um número limitado de exposições e está ligado à memória para itens específicos, enquanto a aprendizagem de habilidades tende a exigir múltiplas exposições e não depende da lembrança de elementos específicos^(2,5)^.

Outras manifestações de memória implícita também foram descritas, incluindo efeitos enviesadores de experiências anteriores sobre julgamentos perceptivos, cognitivos, afetivos e sociais^(2)^. Tais efeitos ilustram a amplitude dos domínios nos quais a memória implícita pode atuar.

Na Fonoaudiologia, ciência voltada ao estudo, prevenção, avaliação e tratamento das alterações da comunicação como a linguagem, a fala, a voz e a audição, muitos comportamentos clínicos e terapêuticos envolvem processos que ocorrem de maneira automática e inconsciente. A aquisição e a modificação de padrões comunicativos frequentemente resultam de práticas repetitivas, ajustes sensório-motores e aprendizagens incidentais, características típicas de processos implícitos^(6-11)^.

Apesar de a memória implícita ser frequentemente mobilizada de forma indireta em práticas avaliativas e terapêuticas fonoaudiológicas, observa-se a ausência de uma sistematização que identifique como esse construto tem sido conceituado, operacionalizado e aplicado empiricamente no campo. Ainda é pouco evidente quais áreas da Fonoaudiologia têm incorporado a memória implícita, quais delineamentos e tarefas têm sido utilizados para avaliá-la, e quais objetivos clínicos ou investigativos têm orientado seu uso. Essa lacuna dificulta tanto a consolidação teórica do tema quanto a tradução do conhecimento produzido para a prática clínica baseada em evidências. Dessa forma, esta revisão teve como objetivo mapear como a memória implícita tem sido abordada na produção científica internacional em Fonoaudiologia.

## MÉTODO

Trata-se de uma revisão de escopo fundamentada no manual Joanna Briggs Institute (JBI)^(12)^ e nas recomendações do Preferred Reporting Items for Systematic reviews and Meta-Analyses extension for Scoping Reviews (PRISMA-ScR)^(13)^ (Figura 1). O protocolo desta revisão foi registrado na plataforma Open Science Framework (10.17605/OSF.IO/PNDWF).

**Figura 1:** Fluxograma PRISMA

Fonte: Baseado em Page et al.^(13)^

Foram definidas as seguintes etapas para este estudo: identificação da questão da pesquisa; estabelecimento dos critérios de elegibilidade alinhados à questão de investigação e ao objetivo; elaboração da estratégia de busca; identificação dos estudos relevantes; seleção dos estudos; extração dos dados; e mapeamento dos dados e sumarização dos resultados obtidos. Ademais, foi realizada a avaliação do nível de evidência dos estudos.

### Questão de investigação

Com base no acrónimo PCC (população, conceito e contexto), em que a população é a fonoaudiologia e o conceito é a memória implícita, foi definida a seguinte questão de investigação: “O que tem sido estudado sobre memória implícita na fonoaudiologia?”. Os autores optaram por não utilizar descritores específicos para o contexto de modo a ampliar os resultados.

### Critério de elegibilidade

Foram incluídos estudos publicados em português, inglês e espanhol, sem restrição quanto ao período de publicação. Excluíram-se estudos cujo acesso estivesse condicionado a pagamento ou que não estivessem disponíveis na íntegra, mesmo após tentativa de contato com os autores (ressalta-se que parte significativa dos periódicos foi acessada gratuitamente por meio da rede institucional da Universidade de São Paulo, Brasil). Também foram excluídos documentos oriundos da literatura cinzenta (como resumos de congressos e anais de eventos, capítulos de livros, dissertações e teses), bem como cartas ao editor, artigos de opinião e resenhas.

A partir dos textos incluídos, foram analisadas as suas respectivas referências e foram incluídos os textos que se enquadraram ao tema de pesquisa, assim como os demais critérios citados anteriormente.

### Estratégias de buscas

As palavras-chaves e os descritores utilizados para a busca foram identificados nos *Descritores de Ciência e Saúde (DeCS)* e no *Medical Subject Headings (MeSH)*, bem como por buscas prévias na literatura (Tabela 1).

**Tabela 1:** Descritores encontrados em pesquisas automáticas e manuais

Speech-language therapy	"Voice Quality" OR "Qualities, Voice" OR "Quality, Voice" OR "Voice Qualities" OR "Speech-Language Pathology" OR "Language Pathology" OR "Pathology, Language" OR "Pathology, Speech" OR "Pathology, Speech Language" OR "Pathology, Speech-Language" OR "Speech and Hearing Science" OR "Speech and Hearing Studies" OR "Speech and Hearing Study" OR "Speech and Language Pathology and Audiology" OR "Audiology" OR "Speech Language Pathology" OR "Speech Pathology" OR "Speech, Language and Hearing Pathology" OR "Speech, Language and Hearing Sciences" OR "Speech, Language and Hearing Studies" OR "Speech, Language and Hearing Study" OR "Speech-Language Pathology and Audiology" OR "Speech-Language-Hearing Pathology"
Implicit memory	memory OR memories OR "implicit memory" OR "memory, implicit" OR "Repetition Priming" OR "Priming, Repetition" OR "Bias, Implicit" OR "Bias, Hidden" OR "Bias, Subconscious" OR "Bias, Unconscious" OR "Hidden Bias" OR "Implicit Bias" OR "Subconscious Bias" OR "Unconscious Bias" OR "Implicit association test"

Fonte: os autores

Com base nos descritores definidos, foi realizada uma nova busca nas bases de dados *Medline, Biblioteca Virtual em Saúde (BVS), Excerpta Medica Database (EMBASE), Web of Science, SciVerse Scopus (SCOPUS), Cumulative Index to Nursing and Allied Health Literature (CINAHL)*, em 15 de abril de 2025. As estratégias de busca foram adaptadas para cada base de dados com a utilização dos operadores booleanos AND e OR (Tabela 2).

**Tabela 2:** Estratégia de busca

Medline	(memory[Text Word] OR memories[Text Word] OR "implicit memory"[Text Word] OR "memory, implicit"[Text Word] OR "Repetition Priming"[Text Word] OR "Priming, Repetition"[Text Word] OR "Bias, Implicit"[Text Word] OR "Bias, Hidden"[Text Word] OR "Bias, Subconscious"[Text Word] OR "Bias, Unconscious"[Text Word] OR "Hidden Bias"[Text Word] OR "Implicit Bias"[Text Word] OR "Subconscious Bias"[Text Word] OR "Unconscious Bias"[Text Word] OR "Implicit association test"[Text Word]) AND ("Voice Quality"[Text Word] OR "Qualities, Voice"[Text Word] OR "Quality, Voice"[Text Word] OR "Voice Qualities"[Text Word] OR "Speech-Language Pathology"[Text Word] OR "Language Pathology"[Text Word] OR "Pathology, Language"[Text Word] OR "Pathology, Speech"[Text Word] OR "Pathology, Speech Language"[Text Word] OR "Pathology, Speech-Language"[Text Word] OR "Speech and Hearing Science"[Text Word] OR "Speech and Hearing Studies"[Text Word] OR "Speech and Hearing Study"[Text Word] OR "Speech and Language Pathology and Audiology"[Text Word] OR "Audiology"[Text Word] OR "Speech Language Pathology"[Text Word] OR "Speech Pathology"[Text Word] OR "Speech, Language and Hearing Pathology"[Text Word] OR "Speech, Language and Hearing Sciences"[Text Word] OR "Speech, Language and Hearing Studies"[Text Word] OR "Speech, Language and Hearing Study"[Text Word] OR "Speech-Language Pathology and Audiology"[Text Word] OR "Speech-Language-Hearing Pathology"[Text Word])
BVS	(memory OR memories OR "implicit memory" OR "memory, implicit" OR "Repetition Priming" OR "Priming, Repetition" OR "Bias, Implicit" OR "Bias, Hidden" OR "Bias, Subconscious" OR "Bias, Unconscious" OR "Hidden Bias" OR "Implicit Bias" OR "Subconscious Bias" OR "Unconscious Bias" OR "Implicit association test" ) AND ("Voice Quality" OR "Qualities, Voice" OR "Quality, Voice" OR "Voice Qualities" OR "Speech-Language Pathology" OR "Language Pathology" OR "Pathology, Language" OR "Pathology, Speech" OR "Pathology, Speech Language" OR "Pathology, Speech-Language" OR "Speech and Hearing Science" OR "Speech and Hearing Studies" OR "Speech and Hearing Study" OR "Speech and Language Pathology and Audiology" OR "Audiology" OR "Speech Language Pathology" OR "Speech Pathology" OR "Speech, Language and Hearing Pathology" OR "Speech, Language and Hearing Sciences" OR "Speech, Language and Hearing Studies" OR "Speech, Language and Hearing Study" OR "Speech-Language Pathology and Audiology" OR "Speech-Language-Hearing Pathology") AND NOT db:("MEDLINE") AND instance:"regional" AND instance:"regional"
EMBASE	(memory OR memories OR 'implicit memory' OR 'memory, implicit' OR 'repetition priming' OR 'priming, repetition' OR 'bias, implicit' OR 'bias, hidden' OR 'bias, subconscious' OR 'bias, unconscious' OR 'hidden bias' OR 'implicit bias' OR 'subconscious bias' OR 'unconscious bias' OR 'implicit association test') AND ('voice quality' OR 'qualities, voice' OR 'quality, voice' OR 'voice qualities' OR 'speech-language pathology' OR 'language pathology' OR 'pathology, language' OR 'pathology, speech' OR 'pathology, speech language' OR 'pathology, speech-language' OR 'speech and hearing science' OR 'speech and hearing studies' OR 'speech and hearing study' OR 'speech and language pathology and audiology' OR 'audiology' OR 'speech language pathology' OR 'speech pathology' OR 'speech, language and hearing pathology' OR 'speech, language and hearing sciences' OR 'speech, language and hearing studies' OR 'speech, language and hearing study' OR 'speech-language pathology and audiology' OR 'speech-language-hearing pathology') AND [embase]/lim NOT ([embase]/lim AND [medline]/lim)
Web of Science	ALL=(memory OR memories OR "implicit memory" OR "memory, implicit" OR "Repetition Priming" OR "Priming, Repetition" OR "Bias, Implicit" OR "Bias, Hidden" OR "Bias, Subconscious" OR "Bias, Unconscious" OR "Hidden Bias" OR "Implicit Bias" OR "Subconscious Bias" OR "Unconscious Bias" OR "Implicit association test" ) AND ALL=("Voice Quality" OR "Qualities, Voice" OR "Quality, Voice" OR "Voice Qualities" OR "Speech-Language Pathology" OR "Language Pathology" OR "Pathology, Language" OR "Pathology, Speech" OR "Pathology, Speech Language" OR "Pathology, Speech-Language" OR "Speech and Hearing Science" OR "Speech and Hearing Studies" OR "Speech and Hearing Study" OR "Speech and Language Pathology and Audiology" OR "Audiology" OR "Speech Language Pathology" OR "Speech Pathology" OR "Speech, Language and Hearing Pathology" OR "Speech, Language and Hearing Sciences" OR "Speech, Language and Hearing Studies" OR "Speech, Language and Hearing Study" OR "Speech-Language Pathology and Audiology" OR "Speech-Language-Hearing Pathology" )
SCOPUS	( TITLE-ABS-KEY ( memory OR memories OR "implicit memory" OR "memory, implicit" OR "Repetition Priming" OR "Priming, Repetition" OR "Bias, Implicit" OR "Bias, Hidden" OR "Bias, Subconscious" OR "Bias, Unconscious" OR "Hidden Bias" OR "Implicit Bias" OR "Subconscious Bias" OR "Unconscious Bias" OR "Implicit association test" ) AND TITLE-ABS-KEY ( "Voice Quality" OR "Qualities, Voice" OR "Quality, Voice" OR "Voice Qualities" OR "Speech-Language Pathology" OR "Language Pathology" OR "Pathology, Language" OR "Pathology, Speech" OR "Pathology, Speech Language" OR "Pathology, Speech-Language" OR "Speech and Hearing Science" OR "Speech and Hearing Studies" OR "Speech and Hearing Study" OR "Speech and Language Pathology and Audiology" OR "Audiology" OR "Speech Language Pathology" OR "Speech Pathology" OR "Speech, Language and Hearing Pathology" OR "Speech, Language and Hearing Sciences" OR "Speech, Language and Hearing Studies" OR "Speech, Language and Hearing Study" OR "Speech-Language Pathology and Audiology" OR "Speech-Language-Hearing Pathology" ) )
CINAHL	(memory OR memories OR "implicit memory" OR "memory, implicit" OR "Repetition Priming" OR "Priming, Repetition" OR "Bias, Implicit" OR "Bias, Hidden" OR "Bias, Subconscious" OR "Bias, Unconscious" OR "Hidden Bias" OR "Implicit Bias" OR "Subconscious Bias" OR "Unconscious Bias" OR "Implicit association test") AND ("Voice Quality" OR "Qualities, Voice" OR "Quality, Voice" OR "Voice Qualities" OR "Speech-Language Pathology" OR "Language Pathology" OR "Pathology, Language" OR "Pathology, Speech" OR "Pathology, Speech Language" OR "Pathology, Speech-Language" OR "Speech and Hearing Science" OR "Speech and Hearing Studies" OR "Speech and Hearing Study" OR "Speech and Language Pathology and Audiology" OR "Audiology" OR "Speech Language Pathology" OR "Speech Pathology" OR "Speech, Language and Hearing Pathology" OR "Speech, Language and Hearing Sciences" OR "Speech, Language and Hearing Studies" OR "Speech, Language and Hearing Study" OR "Speech-Language Pathology and Audiology" OR "Speech-Language-Hearing Pathology")

Fonte: os autores

### Identificação e seleção dos estudos relevantes / extração dos dados

Os dados foram exportados e categorizados na plataforma *Rayyan Qatar Computing Research Institute* (QCRI)^(14)^. A primeira leitura foi realizada por dois pesquisadores independentes. Um terceiro avaliador foi convidado para auxiliar na tomada de decisões (inclusão ou exclusão) em casos de divergência. Assim, os estudos selecionados nessa etapa foram submetidos à leitura na íntegra, permitindo uma análise de sua relevância quanto à sua inclusão na revisão.

Ressalta-se que foram elegíveis para inclusão os textos que abordassem a memória implícita de forma direta ou indireta, seja em seus descritores, títulos, resumos, objetivos, métodos e/ou resultados. Essa estratégia permitiu uma avaliação conceitual aprofundada do conteúdo dos artigos, garantindo a inclusão de evidências pertinentes ao fenômeno investigado, mesmo na ausência de terminologia padronizada nos vocabulários controlados ou nos próprios descritores dos estudos.

A extração dos dados foi realizada por meio de uma ficha padronizada elaborada pelos autores. Foram extraídos o título do artigo, os autores, o ano e o país de publicação, o tipo de estudo, as áreas e os temas prevalentes.

### Avaliação do nível de evidência dos estudos

Os artigos selecionados foram classificados de acordo com seus respectivos níveis de evidência, com base nos critérios estabelecidos pela *Oxford Centre for Evidence-Based Medicine* ^(15)^.

## RESULTADOS

Foram inicialmente identificados 2.565 documentos, dos quais 621 foram excluídos por duplicidade. Os 1.944 registros restantes foram submetidos à leitura de títulos e resumos, resultando na exclusão de 1.895 documentos. Dos 49 documentos selecionados para leitura na íntegra, 19 foram excluídos por não cumprirem com os critérios de elegibilidade e 5 por não estarem disponíveis. Destaca-se que seis estudos que não estavam inicialmente disponíveis na íntegra foram obtidos por solicitação direta aos autores via e-mail.

Ao final dessa etapa, 25 estudos foram considerados elegíveis para inclusão. Em seguida, os revisores realizaram uma busca complementar por meio da análise das listas de referências desses artigos, identificando 21 documentos potencialmente relevantes. Desses, 5 foram excluídos por duplicidade e 2 por indisponibilidade de acesso, resultando em 14 estudos adicionais incluídos. Dessa forma, 39^(16-54)^ estudos compuseram esta revisão de escopo, conforme ilustrado na Figura 1.

### Caracterização dos estudos

Os artigos foram publicados entre os anos de 2003 e 2024 em 9 países, sendo 28^(16-20,22,24,26,28,29,31,32,34,36-41,45-50,52-54)^ provenientes de autores dos Estados Unidos da América, 4^(21,25,27,43)^ da Suécia, 1^(33)^ da Austrália, 1^(23)^ da Bélgica, 1^(42)^ do Brasil, 1^(51)^ do Canadá, 1^(44)^ da Finlândia, 1^(35)^ do Reino Unido e 1^(30)^ de Taiwan. A Tabela 3 traz a caracterização dos estudos por temas e metodologia empregada.

**Tabela 3:** Caracterização dos estudos

**Artigo**	**Autores/ ano**	**País**	**Objetivo do estudo**	**População**	**Tipo de estudo**	**Principais resultados**	**Relevância para revisão**
A1^(16)^	Hopper, 2003	USA	Discutir a aprendizagem implícita no Alzheimer, seguida de um resumo da realidade da implementação de programas terapêuticos em instituições de longa permanência.	Indivíduos com doença de Alzheimer em ambientes de cuidados de longa duração	Teórico/Revisão	A aprendizagem implícita pode estar preservada em indivíduos com demência, mesmo com prejuízos na memória explícita. Técnicas como exposição repetida e aprendizagem sem erro mostram bons resultados. O fonoaudiólogo deve atuar de forma precoce e preventiva, com foco funcional, incluindo triagens e reavaliações contínuas. A intervenção envolve também orientação a cuidadores e adaptação de estratégias conforme a progressão da doença.	Linguagem adulto
A2^(17)^	Anderson e Conture, 2004	USA	Utilizar uma versão adequada à idade do paradigma de priming da estrutura sintática para avaliar experimentalmente as habilidades de processamento sintático de crianças que gaguejam e crianças que não gaguejam.	Crianças que gaguejam e crianças que não gaguejam.	Experimental	Os principais resultados indicaram que crianças que gaguejam apresentaram SRTs mais lentos na ausência de frases de priming e maiores efeitos de priming sintático do que crianças que não gaguejam.	Distúrbios da fluência
A3^(18)^	Turkstra e Bourgeois, 2005	USA	Descrever uma técnica de terapia da memória, a Recuperação Espaçada (RE), projetada para capitalizar os processos de memória processual preservados.	Indivíduo que sofreu traumatismo cranioencefálico com amnésia anterógrada profunda que recebeu terapia de RE por telefone.	Estudo de caso	Os resultados positivos apoiam estudos futuros sobre TCE e sugerem a consideração do papel dos erros na terapia da fala e da linguagem.	Linguagem adulto
A4^(19)^	Tomblin et al., 2007	USA	Testar a hipótese dos déficits na aprendizagem procedural examinando o aprendizado de tempo de reação serial em adolescentes com e sem comprometimento específico de linguagem (CEL).	Adolescentes com e sem CEL	Experimental	Ambos os grupos melhoraram na tarefa de aprendizagem implícita (SRT), mas adolescentes com transtorno específico de linguagem (CEL) apresentaram taxas de aprendizagem mais lentas. Essa diferença foi observada quando o comprometimento foi definido por gramática, mas não por vocabulário.	Linguagem infantil
A5^(20)^	Davis et al., 2009	USA	Introduzir uma nova abordagem para o tratamento da apraxia de fala (AOS) com afasia por meio do uso de manipulação implícita de fonemas por meio de tarefas de rima, omissão e aliteração.	sujeito com apraxia de fala (AOS) e afasia leve	Experimental	O estudo mostrou melhora significativa na produção da fala em crianças, com efeitos pequenos a médios para sons treinados (/ʃ/, /ʤ/, /s/). Houve generalização para palavras não treinadas (T-MAC: 68% para 87%) e melhora perceptível em prosódia e precisão articulatória, confirmada por avaliação clínica de fonoaudiólogos.	Linguagem adulto
A6^(21)^	Hedenius et al., 2011	Sweden	O presente estudo examina a consolidação e a aprendizagem de sequências procedurais a longo prazo em crianças com CEL.	Crianças com Comprometimento Específico da Linguagem (CEL) e crianças com desenvolvimento típico (DT)	Experimental	Embora ambos os grupos tenham mostrado evidências de aprendizagem de sequências iniciais, apenas as crianças com DT mostraram sinais claros de consolidação, embora os dois grupos não tenham diferido na aprendizagem a longo prazo. Quando as crianças foram recategorizadas com base em déficits gramaticais em vez de déficits linguísticos mais amplos, um padrão mais claro emergiu. Enquanto tanto o grupo com comprometimento gramatical quanto o grupo com gramática normal apresentaram evidências de aprendizagem sequencial inicial, apenas aqueles com gramática normal apresentaram consolidação e aprendizagem a longo prazo. De fato, o grupo com comprometimento gramatical pareceu perder qualquer conhecimento sequencial adquirido durante a sessão inicial de teste. Esses achados se mantiveram mesmo quando se controlou o vocabulário ou uma medida ampla de linguagem não gramatical, nenhum dos quais estava associado à memória procedural. Quando a gramática foi examinada como uma variável contínua em todas as crianças, foram observadas as mesmas relações entre memória procedural e gramática, mas não o vocabulário ou a medida mais ampla de linguagem.	Linguagem infantil
A7^(22)^	Leonard, 2011	USA	Apresentar um tutorial sobre priming estrutural e sua relevância para o estudo do desenvolvimento gramatical e intervenção na linguagem.	Não se aplica	Teórico/Revisão	Evidências da literatura sobre priming estrutural fornecem insights sobre a transição do uso gramatical conservador inicial para o uso gramatical abstrato mais amplo em crianças pequenas e sugerem maneiras pelas quais as atividades de intervenção da linguagem podem ser modificadas para promover maiores mudanças gramaticais em crianças com dificuldades de linguagem.	Linguagem infantil
A8^(23)^	Gabriel et al., 2012	Belgium	O objetivo deste estudo é avaliar a Hipótese do Déficit Processual em crianças com Comprometimento Específico de Linguagem (CEL) utilizando o paradigma Serial Reaction Time (SRT), estabelecendo uma situação experimental caracterizada por mais tentativas de aprendizagem do que em estudos anteriores e utilizando uma sequência de aprendizagem que, mais especificamente, testaria a capacidade das crianças de aprender regularidades estatísticas complexas (ou seja, de segunda ordem) dentro da sequência.	Quinze crianças com CEL e seus pares com desenvolvimento típico (DT)	Experimental	Embora no Experimento 1 as crianças com CEL tenham demonstrado aprendizagem, elas foram mais lentas e cometeram mais erros do que seus pares com DT. No entanto, essas fraquezas relativas desapareceram quando a natureza do modo de resposta mudou (Experimento 2).	Linguagem infantil
A9^(24)^	Silkes et al., 2012	USA	Este estudo de Fase I foi realizado para investigar a eficácia potencial do priming de repetição mascarada como tratamento para anomia.	Sujeito com afasia moderada a grave não fluente	Experimental	Os resultados revelaram um pequeno efeito do treinamento na nomeação de itens treinados em uma categoria, bem como um padrão de melhora na outra categoria que não atendeu totalmente ao critério de um efeito pequeno. Houve também um efeito médio de generalização entre categorias, embora nenhum efeito de generalização tenha sido observado para itens não treinados dentro de categorias semânticas. Melhorias foram observadas em algumas medidas da função geral da linguagem.	Linguagem adulto
A10^(25)^	Rönnberg et al., 2013	Sweden	Este artigo examina o modelo de Facilidade de Compreensão da Linguagem à luz de novas descobertas comportamentais e neurais sobre o papel da capacidade de memória de trabalho (WMC) no processamento da linguagem unimodal e bimodal.	Não se aplica	Teórico/Revisão	O novo modelo ELU é um sistema de predição de significado que depende de interações fonológicas e semânticas em mecanismos de processamento implícito rápido e explícito mais lento, que dependem da WMC, embora de maneiras diferentes. Baseia-se em descobertas que abordam a relação entre a WMC e (a) processos de atenção precoce na audição da fala, (b) processamento de sinais em aparelhos auditivos e seus efeitos na memória de curto prazo, (c) inibição de mascaradores de fala e seu efeito na memória episódica de longo prazo, (d) os efeitos da deficiência auditiva na memória episódica e semântica de longo prazo e, finalmente, (e) esforço auditivo.	Audiologia
A11^(26)^	Valitchka e Turkstra, 2013	USA	Descrever os padrões de comunicação entre pacientes internados com deficiências de memória declarativa e membros da equipe de reabilitação durante o estágio inicial após a lesão.	Cinco adultos com lesão cerebral adquirida e comprometimento da memória declarativa.	Estudo transversal	Os resultados mostraram que os funcionários e visitantes frequentemente faziam perguntas declarativas aos participantes, cujas respostas não eram verificáveis (por exemplo, perguntas sobre eventos anteriores à lesão). As respostas que podiam ser verificadas muitas vezes estavam incorretas, mas eram aceitas pelos funcionários como corretas. Os resultados sugerem que os funcionários de reabilitação aguda podem precisar de treinamento para se comunicar com pacientes com deficiências de memória declarativa.	Linguagem adulto
A12^(27)^	Hedenius et al., 2013	Sweden	Examinar o aprendizado de sequências procedimentais em DD além de uma única sessão de prática.	Doze crianças com dislexia do desenvolvimento e 17 crianças de controle com desenvolvimento típico	Experimental	Os resultados sugerem que o comprometimento de aprendizagem procedural mais pronunciado na DD pode surgir somente após prática prolongada, em estágios de aprendizagem além de uma única sessão de prática.	Linguagem infantil
A13^(28)^	Farias et al., 2014	USA	O presente estudo visa promover a compreensão e a validade de um novo tratamento para a Apraxia de fala, a manipulação implícita de fonemas.	Indivíduo com apraxia de fala	Experimental	Comportamentais: A resposta ao treinamento implícito de combinações consonantais complexas resultou em melhorias que se mantiveram 6 semanas após o tratamento. Além disso, este tratamento foi generalizado para combinações consonantais mais simples em palavras. Imagem: Imagens funcionais durante a manipulação implícita de fonemas mostraram ativação significativa em regiões cerebrais responsáveis pelo processamento fonológico, em comparação com a tarefa semântica de base.	Linguagem adulto
A14^(29)^	Moring et al., 2014	USA	Investigar se as medidas atuais para o zumbido são suscetíveis a um aumento do erro devido ao priming e, em caso afirmativo, examinar a viabilidade de usar o Teste de Associação Implícita para uma medição alternativa do sofrimento relacionado ao zumbido.	Pessoas com zumbido	Experimental	Embora os participantes com zumbido não tenham considerado as palavras relacionadas ao som significativamente mais negativas e as pontuações do TAI não tenham previsto as pontuações no Inventário de Incapacidade por Zumbido, o priming afetou as atitudes implícitas negativas em relação às palavras relacionadas ao som.	Audiologia
A15^(30)^	Hsu e Bishop, 2014	Taiwan	Testar a hipótese de déficit procedural do comprometimento específico da linguagem (CEL) comparando o desempenho de crianças em duas tarefas de aprendizagem procedural motora e uma tarefa de aprendizagem de sequência verbal implícita.	Crianças de 7 a 11 anos com CEL (n = 48), crianças com desenvolvimento típico e idade correspondente (n = 20) e crianças mais novas com desenvolvimento típico e gramática receptiva correspondente (n = 28).	Experimental	Em uma tarefa de tempo de reação serial, as crianças com CEL tiveram o mesmo nível de desempenho das crianças com gramática correspondente, mas foram piores do que os controles com idade correspondente na aprendizagem de sequências motoras. Quando testadas com uma tarefa de aprendizagem procedural motora que não envolvia a aprendizagem de relações sequenciais entre elementos discretos (por exemplo, rotor de perseguição), as crianças com CEL tiveram um desempenho comparável ao de crianças com idade correspondente e melhor do que os controles com gramática correspondente mais jovens. Além disso, foi encontrada aprendizagem implícita ruim de sequências de palavras em uma tarefa de memória verbal (efeito Hebb) nas crianças com CEL.	Linguagem infantil
A16^(31)^	Silkes, 2015	USA	Investigar o efeito da exposição repetida a estímulos de identidade mascarados combinados com imagens ao longo de várias tentativas, sessões e dias sobre a capacidade das pessoas com anomia de nomear essas imagens.	Quatro participantes com anomia	Experimental	Todos os participantes apresentaram algum progresso na identificação dos itens treinados, embora em graus variados, e os itens treinados (preparados) geralmente apresentaram uma melhora maior do que os itens não treinados vistos o mesmo número de vezes. A generalização entre categorias foi observada em alguns participantes, mas pouca ou nenhuma generalização dentro da categoria ocorreu. Mudanças mínimas ocorreram nas medidas de habilidade linguística geral.	Linguagem adulto
A17^(32)^	Lee e Man, 2017	USA	Examinar a viabilidade e eficácia do tratamento de preparação estrutural implícita em um indivíduo com afasia agramática.	Indivíduo com afasia	Estudo de caso	MJ apresentou melhora significativa na produção de frases em dativas preposicionais, tanto treinadas quanto não treinadas, ao longo das sessões de treinamento. É importante ressaltar que esses efeitos foram mantidos após quatro semanas de acompanhamento sem intervenção. Melhora notável também foi observada na complexidade sintática da produção da fala conectada, como o aumento da produção de verbos e frases lexicais.	Linguagem adulto
A17^(33)^	Calder et al., 2017	Austrália	Este estudo investigou a eficácia de técnicas combinadas de intervenção explícita e implícita, aplicadas por um fonoaudiólogo, para melhorar a gramática receptiva e expressiva	Três crianças (dois meninos; uma menina) com idades entre 6;2–7;0, apresentando dificuldades gramaticais	Experimental	Dois dos três participantes obtiveram ganhos estatisticamente significativos em testes padronizados de gramática geral receptiva e expressiva. Duas das três crianças obtiveram melhora estatisticamente significativa nas medidas de morfossintaxe expressiva, com um participante continuando a melhorar cinco semanas após o tratamento.	Linguagem infantil
A19^(34)^	Walden e Lesner, 2018	USA	Este estudo avaliou as atitudes implícitas e explícitas em relação às pessoas que gaguejam entre jovens adultos com fluência normal.	adultos sem gagueira, ou com fluência típica, em direção à fala gaguejada	Estudo transversal	Os resultados corroboraram a existência de um estereótipo negativo da gagueira. Os participantes demonstraram atitudes negativas implícitas e explícitas em relação às pessoas que gaguejam. Atitudes explícitas em relação aos gaguejantes, mas não atitudes implícitas, foram significativamente previstas pelos escores de desejabilidade social. A familiaridade com a gagueira foi significativamente associada a atitudes implícitas, mas não explícitas, em relação à gagueira.	Distúrbios da fluência
A20^(35)^	Garraffa et al., 2018	Reino Unido	Investigar se um grupo de crianças com Transtorno do Desenvolvimento da Aprendizagem apresentou comprometimento na aprendizagem implícita da sintaxe (priming sintático) após experiências sintáticas individuais, e o curso temporal de tais efeitos.	Crianças de cinco a seis anos de idade, falantes de italiano, com Transtorno do Desenvolvimento da Aprendizagem e controles com desenvolvimento típico, pareados por idade e linguagem	Experimental	Crianças com Transtorno do Desenvolvimento da Aprendizagem apresentaram priming sintático imediato, mas menos intenso e menos duradouro do que crianças com desenvolvimento típico da mesma idade. O efeito não se manteve após frases intermediárias, e não houve efeito de priming cumulativo em nenhum grupo.	Linguagem infantil
A21^(36)^	Lee et al., 2019	USA	Examinar se pessoas com afasia apresentam efeitos de priming estrutural preservados e duradouros durante a interpretação de frases sintaticamente ambíguas e se os efeitos de priming ocorrem independentemente ou em conjunto com informações lexicais (verbais)	Pessoas com afasia e idosos saudáveis	Experimental	Após ler uma frase primária com uma interpretação específica, idosos saudáveis e pessoas com afasia tenderam a interpretar uma PP ambígua em uma frase alvo da mesma maneira e com tempos de resposta mais rápidos. É importante ressaltar que ambos os grupos continuaram a apresentar esse efeito de priming durante um intervalo (Experimento 2), embora o efeito não tenha sido tão confiável em termos de tempos de resposta. No entanto, nenhum dos grupos apresentou aumento lexical (específico do verbo) no priming, desviando-se do forte aumento lexical observado em jovens adultos de estudos anteriores.	Linguagem adulto
A22^(37)^	Man et al., 2019	USA	Investigar se o priming lexicalmente independente (estrutural abstrato) e/ou o priming lexicalmente específico (verbo) produz uma facilitação imediata e mais duradoura da produção sintática em pessoas com PWA.	Dezessete indivíduos com afasia (PWA) and 20 idosos saudáveis	Experimental	Idosos saudáveis ​​demonstraram priming abstrato em transitivos e dativos, não apenas na condição de priming imediato (Experimento 1), mas também na condição de priming duradouro (Experimento 2). Eles também demonstraram priming significativamente aumentado pela sobreposição verbal (reforço lexical) em transitivos durante o priming imediato. Pessoas com AWP demonstraram priming abstrato em transitivos tanto em condições de priming imediato quanto duradouro. No entanto, a magnitude do priming não foi aumentada pela sobreposição verbal.	Linguagem adulto
A23^(38)^	Kamhi, 2019	USA	Analisa como o desenvolvimento da fala e da linguagem pode ser visto como proceduralização e aprendizagem de habilidades.	Indivíduos com distúrbios da fala e da linguagem.	Teórico/Revisão	Medidas com referência a normas fornecem uma boa medida dos processos deliberativos de fala e linguagem, enquanto conversas e discursos sociais sobre tópicos familiares com ouvintes familiares fornecem a melhor indicação de processos proceduralizados de fala e linguagem. Os objetivos e o planejamento da intervenção precisam considerar quais operações de fala e linguagem devem ser proceduralizadas e quais exigirão principalmente processamento deliberativo. Pesquisas adicionais e a aplicação clínica dessas ideias são claramente necessárias para desenvolver critérios mais precisos que definam a linha de demarcação entre processos deliberativos e proceduralizados de fala e linguagem.	Linguagem infantil
A24^(39)^	Walden et al., 2020	USA	Avaliaram atitudes implícitas e explícitas em relação à gagueira e aqueles que gaguejam entre fonoaudiólogos.	fonoaudiólogos	Transversal	Como grupo, os clínicos demonstraram atitudes implícitas negativas em relação à gagueira. Atitudes explícitas em relação a uma pessoa que gagueja foram positivas, embora menos positivas do que as atitudes em relação a uma pessoa que não gagueja. A quantidade de exposição prévia à gagueira entre esses fonoaudiólogos experientes não foi significativamente associada a atitudes implícitas ou explícitas.	Distúrbios da fluência
A25^(40)^	Wada et al., 2020	USA	Examinar o uso de priming estrutural combinado com um procedimento de reformulação focado para obter orações relativas centradas no sujeito e no objeto, de alunos com transtornos do desenvolvimento da linguagem (TDL) e alunos com desenvolvimento típico (DT).	26 crianças (13 TLD, 13 DT), com idades entre 6;10 e 10;11 (anos; meses)	Experimental	Crianças com TDL e com desenvolvimento típico foram mais precisas na produção de orações relativas com foco no sujeito do que no objeto. No entanto, crianças com TDL precisaram de mais tentativas para alcançar desempenho consistente em ambos os tipos de estruturas, indicando uma taxa de aprendizagem mais lenta.	Linguagem infantil
A26^(41)^	Evans, 2021	USA	Discutir o conceito de viés implícito na área da audiologia.	Não se aplica	Teórico/Revisão	O artigo discute preconceitos e vieses implícitos na prática audiológica, incluindo a sub-representação de profissionais negros, o viés socioeconômico e linguístico, e o audismo. Destaca como esses fatores afetam a forma como informações são compartilhadas com famílias, e propõe uma abordagem afirmativa e culturalmente sensível na comunicação clínica.	Audiologia
A27^(42)^	Maziero et al., 2021	Brasil	Comparar o desempenho de indivíduos com comprometimento cognitivo leve (CCL) com o de idosos saudáveis ​​em uma tarefa de compreensão de inferência textual e determinar os melhores preditores de desempenho nessa habilidade considerando uma tarefa de memória episódica verbal e duas tarefas semânticas.	Indivíduos com comprometimento cognitivo leve e idosos saudáveis	Estudo transversal	Pacientes com CCL apresentaram desempenho inferior ao de idosos saudáveis, e não houve diferenças entre os subgrupos com CCL (amnésicos e não amnésicos). Os melhores preditores para a realização de inferências foram a memória verbal no grupo com CCL e tarefas semânticas no grupo sem CCL.	Linguagem adulto
A28^(43)^	Hedenius et al., 2021	Sweden	examinar se a prática inicial prolongada na Sessão 1 normalizaria a consolidação procedural em crianças com Dislexia do desenvolvimento.	Crianças com Dislexia do Desenvolvimento e crianças com desenvolvimento típico	Experimental	A quantidade de aprendizagem de sequência foi semelhante nos dois grupos no final da sessão 1 (p=0,797, η ρ 2 = 0,001). Na sessão de acompanhamento após 24 horas, o desempenho no grupo DD foi pior em comparação com o grupo DT (p=0,003, η ρ 2 = 0,129). A quantidade de conhecimento de sequências na Sessão 2 previu uma variância única na fluência da leitura (p=0,024, η ρ 2 = 0,083), independentemente da consciência fonêmica e dos sintomas de desatenção das crianças.	Linguagem infantil
A29^(44)^	Kautto e Mainela-Arnold, 2022	Finland	Déficits na aprendizagem procedural foram hipotetizados para contribuir para a linguagem e em particular dificuldades gramaticais de indivíduos com comprometimento específico de linguagem (CEL). Este estudo testou essa hipótese examinando o aprendizado de tempo de reação serial (SRT) em adolescentes com e sem CELI.	Adolescentes que tinham sido identificados com comprometimento específico de linguagem e adolescentes com linguagem normal.	Experimental	Adolescentes com CELI mostraram taxas de aprendizagem mais lentas durante a aprendizagem de padrões em comparação com os controles. Quando o comprometimento da linguagem foi definido em termos de comprometimentos gramaticais, taxas de aprendizagem mais lentas semelhantes foram encontradas, mas quando o comprometimento da linguagem foi baseado no vocabulário, diferenças de grupo não foram encontradas.	Linguagem infantil
A30^(45)^	Baron e Arbel, 2022	USA	Apresentar evidências da interação entre sistemas de aprendizagem implícita e explícita e destacar as características da intervenção que promovem a aprendizagem implícita ou explícita, bem como medidas de resultados que exploram o conhecimento implícito ou explícito.	Não se aplica	Teórico/Revisão	Muitas características da intervenção (por exemplo, instruções, técnicas de elicitação, feedback) podem ser manipuladas para mover uma intervenção ao longo do continuum implícito-explícito. Dado o viés para o uso de estratégias de aprendizagem explícita que se desenvolve ao longo da infância e na idade adulta, os clínicos devem estar cientes de que a maioria das intervenções (mesmo aquelas que promovem a aprendizagem implícita) envolverá o sistema de aprendizagem explícita. No entanto, uma maior conscientização sobre os sistemas de aprendizagem implícita e explícita e suas demandas cognitivas permitirá que os clínicos escolham a intervenção mais apropriada para o comportamento-alvo.	Linguagem infantil
A31^(46)^	Tabrizi et al., 2022	USA	O priming por repetição tem sido sugerido como um método para tratar processos implícitos no tratamento da anomia. Estudos anteriores utilizaram o priming mascarado para esse fim. Este estudo amplia esse trabalho com priming visível, uma abordagem mais viável clinicamente.	Três participantes com afasia	Experimental	Todos os participantes melhoraram na identificação dos itens treinados imediatamente após o tratamento, com melhorias maiores para os itens treinados do que para os não treinados. Todos os participantes mantiveram algum grau de melhoria nos itens treinados três meses após o tratamento, embora o grau variasse entre os participantes. Ocorreu uma generalização inconsistente para itens não expostos. Foram observadas melhorias em algumas áreas da linguagem mais ampla, embora estas variassem.	Linguagem adulto
A32^(47)^	Norotsky et al., 2026	USA	Entender o papel do preconceito racial implícito nas avaliações perceptivo-auditivas de vozes disfônicas, determinando se existe um efeito de preconceito para ouvintes novatos em suas avaliações perceptivo-auditivas de falantes negros e brancos.	Estudantes de pós-graduação em patologia da fala e da linguagem da Universidade de Boston	Experimental	Tanto falantes negros quanto brancos foram classificados como menos gravemente disfônicos quando sua raça foi rotulada como negra.	Voz
A33^(48)^	Lopez et al., 2023	USA	Determinar como a maneira de aquisição da L2 pode afetar a generalização interlinguística após o tratamento em L2, visando separadamente o léxico (nomeação de objetos) e a gramática (construção de frases)	Dois bilíngues espanhol-inglês com afasia	Experimental	Conforme previsto, P2 apresentou generalização interlínguas após o tratamento verbo/gramática (VNeST), enquanto P1 não. No entanto, ao contrário da previsão de que ambos os participantes deveriam apresentar generalização interlínguas após o tratamento substantivo (SFA), apenas P1 apresentou generalização interlínguas na nomeação de objetos.	Linguagem adulto
A34^(49)^	Silkes, 2023	USA	Ampliar as evidências para um tratamento da anomia baseado no priming de repetição.	Cinco participantes com afasia crônica	Quase-experimental	Itens previamente ativados por priming resultaram em melhorias maiores na nomeação do que aqueles apenas praticados, mas sem priming. Os dados sugerem que os ganhos podem ultrapassar a associação direta com os estímulos treinados. Houve variação nos efeitos de manutenção e generalização.	Linguagem adulto
A35^(50)^	Lee et al., 2024	USA	Examinar o impacto do envelhecimento na força e na longevidade dos efeitos do priming estrutural abstrato e do reforço lexical.	Adultos jovens e idosos	Experimental	No priming imediato, adultos mais velhos apresentaram priming estrutural abstrato e aumento lexical em transitivos e dativos, semelhante ao observado em adultos jovens. No priming de longo prazo, apenas os efeitos do priming abstrato foram significativos em ambos os grupos de participantes, sem evidência de redução do priming relacionada à idade.	Linguagem adulto
A36^(51)^	Beadle et al., 2024	Canadá	Investigar as percepções de adultos mais velhos e mais jovens sobre adultos mais velhos e mais jovens que usam aparelhos auditivos.	Trinta adultos mais velhos e 30 adultos mais jovens, que relataram não ter aparelhos auditivos ou deficiência auditiva diagnosticada.	Transversal	Em ambos os TAIs, participantes mais velhos e mais jovens responderam mais rápido e com mais precisão quando imagens de indivíduos usando aparelhos auditivos foram pareadas com palavras negativas em comparação com palavras positivas. As avaliações das fotos não variaram em relação à presença ou ausência de aparelhos auditivos para nenhuma das faixas etárias.	Audiologia
A37^(52)^	Montgomery et al., 2024	USA	Propomos que a aprendizagem implícita, incluindo a preparação sintática, tem uma promessa terapêutica para melhorar o conhecimento sintático de crianças com transtorno do desenvolvimento da linguagem (TDL).	crianças com transtorno do desenvolvimento da linguagem (TDL)	Teórico/Revisão	A aprendizagem baseada em blocos estatisticamente induzida é um poderoso impulsionador da aprendizagem sintática, e o priming sintático é uma forma dessa aprendizagem. Episódios repetidos de priming durante interações linguísticas cotidianas levam as crianças a criar representações sintáticas abstratas e globais na memória de longo prazo. Oferecemos algumas reflexões sobre uma abordagem de intervenção linguística implícita com o priming sintático como foco.	Linguagem infantil
A38^(53)^	Balthazar e Scott, 2024	USA	Apresentar conceitos-chave que apontam para a importância de direcionar frases complexas para crianças em idade escolar e adolescentes com transtornos do desenvolvimento da linguagem (TDL).	Crianças em idade escolar e adolescentes com TDL	Teórico/Revisão	Estruturas complexas de frases representam um desafio fundamental para alunos com TDL ao lidarem com tarefas acadêmicas e linguísticas específicas da disciplina. Programas de tratamento da complexidade de frases empregam um ou mais métodos de tratamento, incluindo priming, modelagem, reformulação, contextualização, instrução metalinguística e combinação de frases. Embora estudos tenham demonstrado consistentemente uma melhora mensurável na produção de frases complexas em desfechos proximais, independentemente da abordagem de tratamento, evidências de mudanças funcionais duradouras em alunos com TDL permanecem escassas. Incentivamos novos tratamentos que visem a compreensão e a produção de frases complexas em contextos acadêmicos da vida real, na prática clínica e na pesquisa.	Linguagem infantil
A39^(54)^	Morton-Jones e Sund, 2024	USA	Explicar o papel impactante que os fonoaudiólogos e clínicos da voz podem desempenhar na prática clínica diária para promover a equidade na saúde, mitigando os efeitos negativos bem documentados dos preconceitos implícitos pessoais na área da saúde e oferecendo práticas culturalmente responsivas.	fonoaudiólogos e clínicos da voz	Teórico/Revisão	As evidências indicam que o preconceito implícito tem um impacto grave na prática clínica diária e no diálogo. Dentro da complexidade do acesso aos cuidados de saúde, um clínico cria barreiras ou facilitadores que determinam a aceitabilidade e a acessibilidade dos cuidados oferecidos. O preconceito implícito não abordado e a falta de práticas culturalmente responsivas podem reduzir a qualidade dos cuidados e, em última análise, impedir um paciente de procurar serviços de voz comportamental.	Voz

Fonte: os autores.

Dos estudos incluídos, 22^(17,19-21,23,24,27-31,33,35-37,40,43,44,46-48,50)^ eram do tipo experimental. Além disso, foram identificados nove^(16,22,25,38,41,45,52-54)^ estudos teóricos, cinco^(26,34,39,42,51)^ estudos observacionais transversais, dois^(18,32)^ estudos de caso, e um^(49)^ estudo quase-experimental.

As áreas mais prevalentes foram relacionadas à linguagem, totalizando 33^(16-24,26-28,30-40,42-46,48-50,52,53)^ artigos (84,6%), distribuídos entre linguagem adulta (15 artigos)^(16,18,20,24,26,28,31,32,36,37,42,46,48-50)^, linguagem infantil (15 artigos) ^(19,21-23,27,30,33,35,38,40,43-45,52,53)^ e distúrbios da fluência e seus transtornos (3 artigos^(17,34,39)^). Os demais estudos abordaram temas vinculados à voz – 2 artigos^(47,54)^ (5,1%) – e à audiologia – 4 artigos^(25,29,41,51)^ (10,3%).

A figura 2 traz a caracterização dos estudos quanto ao seu nível de evidência científica^(15)^. O nível 1 representa o nível mais elevado de evidência e o 5 o menos elevado.

**Figura 2:** Discriminação dos artigos incluídos com base no nível de evidência da OCEBM

Fonte: Baseado em OCEBM^(15)^

## DISCUSSÃO

De modo geral, os textos abordam a aplicabilidade da memória implícita na atuação fonoaudiológica. Diante da diversidade dos achados, optou-se por organizar a discussão em cinco tópicos, com base nas áreas da Fonoaudiologia mais frequentemente abordadas nos estudos: 1. linguagem (1.1. linguagem adulta, 1.2. linguagem infantil e 1.3. distúrbios da fluência e seus transtornos) 2. audiologia; 3. voz.

### Linguagem

#### Linguagem adulta

Temas relacionados à linguagem no adulto foram recorrentes em 15^(16,18,20,24,26,28,31,32,36,37,42,46,48-50)^ estudos, o que evidencia alta prevalência de estudos que abordaram a memória implícita nessa população. Os estudos se debruçaram sobre a aplicação da aprendizagem implícita em contextos terapêuticos fonoaudiológicos e o uso de tarefas implícitas para avaliação e caracterização de transtornos como a afasia e apraxia de fala, o comprometimento cognitivo leve, a demência/doença de Alzheimer, o comprometimento de memória declarativa e o traumatismo cranioencefálico com amnésia anterógrada profunda.

No estudo de Lee et al.^(36)^, investigou-se se pessoas com afasia apresentam efeitos de priming estrutural preservados e duradouros durante a interpretação de frases sintaticamente ambíguas, e se tais efeitos ocorrem de forma independente ou em conjunto com informações lexicais (verbais). O priming estrutural refere-se à tendência involuntária de um indivíduo repetir uma estrutura sintática previamente ouvida ou lida^(55)^. Os resultados indicaram que pessoas com afasia continuam se beneficiando do priming estrutural abstrato, ou seja, da exposição repetida a estruturas gramaticais, mesmo na ausência de repetição de itens lexicais específicos. Esse mecanismo contribui para a compreensão e retenção da linguagem, revelando-se funcional tanto de forma imediata quanto duradoura.

Esses achados estão em consonância com o estudo de Lee e Man^(32)^, que investigou a eficácia do priming estrutural como intervenção em um indivíduo com afasia agramática. Os resultados sugerem que o uso do priming estrutural implícito pode ser uma abordagem viável para a reabilitação da produção sintática na afasia, promovendo a recuperação global da linguagem a longo prazo por meio do fortalecimento das conexões entre representações linguísticas, baseado na experiência.

Ainda sobre o priming estrutural, Man et al.^(37)^ investigaram se o priming estrutural abstrato e/ou o priming lexicalmente específico (com o mesmo verbo) promovem facilitação imediata e duradoura da produção sintática em pessoas com afasia. Os resultados mostraram que os participantes com afasia apresentaram efeitos de priming estrutural abstrato em frases transitivas, de forma semelhante aos adultos mais velhos saudáveis, tanto em medidas imediatas quanto mantidas. No entanto, diferentemente do grupo controle, o grupo com afasia não se beneficiou do reforço lexical, sugerindo uma limitação no uso de pistas lexicais para apoiar a produção sintática. Já Lee et al.^(50)^ observaram que, em adultos mais velhos, tanto os mecanismos lexicalmente específicos quanto os independentes de priming estrutural permanecem preservados, o que corrobora a ideia de que o priming estrutural reflete um mecanismo de aprendizagem linguística ao longo da vida.

López et al.^(48)^ também investigaram a afasia no contexto do tratamento de linguagem para bilíngues afásicos, com o objetivo de compreender como o modo de aquisição da segunda língua (L2) (explícito ou implícito) pode influenciar a generalização interlinguística após a intervenção realizada em L2. O estudo distinguiu os efeitos do tratamento no nível lexical (nomeação de objetos) e no nível gramatical (construção de frases), analisando dois participantes bilíngues com afasia: P1, que aprendeu L2 de forma explícita, e P2, que adquiriu L2 de forma implícita. Observou-se generalização interlinguística em ambos os casos, mas em diferentes componentes da linguagem. Os resultados sugerem que a aprendizagem naturalista (implícita) de uma segunda língua tende a fortalecer os vínculos gramaticais entre as línguas, enquanto a aprendizagem formal (explícita) promove conexões mais robustas no sistema léxico-semântico.

Outra estratégia utilizada no tratamento da afasia é o priming de repetição mascarada, utilizada por Silkes et al.^(24)^ para intervir na anomia. O priming visual mascarado envolve a apresentação visual de um item principal precedido e/ou seguido por outros estímulos visuais que servem para interromper o reconhecimento consciente do item principal pelo cérebro. A partir desse conceito, o propósito deste estudo de Fase I, com um único participante e múltiplas linhas de base foi investigar se o treinamento repetido com priming de repetição mascarada tem efeitos positivos cumulativos na recuperação lexical, o que apoiaria seu potencial como um tratamento alternativo ou complementar para anomia. Assim, as redes seriam fortalecidas e se desenvolveriam automáticas e inconscientes, com influência limitada ou inexistente do processamento consciente. Os resultados sugeriram que o priming visual mascarado pode provocar mudanças na capacidade de nomeação ao longo do tempo e pode ter potencial como ferramenta para melhorar a recuperação de palavras em indivíduos com anomia. Esses resultados corroboram o estudo da Fase II dos mesmos autores^(31)^.

Enquanto isso, Tabrizi et al.^(46)^ abordaram o priming de repetição visível no tratamento da anomia com a justificativa de ser mais viável clinicamente. O priming visível nesse estudo demonstrou que este tratamento pode aumentar a precisão da nomeação para indivíduos com anomia e pode beneficiar outros aspectos da linguagem. Este estudo concorda com o estudo de Silkes^(49)^, em que a aplicação do priming de repetição em cinco indivíduos com afasia demonstrou que itens previamente ativados por priming resultaram em melhorias maiores na nomeação do que aqueles apenas praticados, mas sem priming. Os dados sugerem que os ganhos podem ultrapassar a associação direta com os estímulos treinados.

A manipulação implícita de fonemas tem sido utilizada como estratégia terapêutica na apraxia de fala. Estudos experimentais com delineamento de linha de base múltipla com sujeito único, conduzidos por Davis et al.^(20)^ e Farias et al.^(28)^, demonstraram resultados positivos com essa abordagem. Os participantes apresentaram melhora na produção da fala espontânea e na precisão das palavras treinadas, incluindo redução de distorções sonoras, diminuição de erros fonológicos e aprimoramento da prosódia. Ambos os estudos também relataram generalização significativa para palavras não tratadas. Além disso, evidências de neuroimagem indicaram que essa abordagem ativa áreas cerebrais envolvidas no processamento fonológico, no planejamento e na programação motora da fala.

O estudo de Hopper^(16)^ discute a aprendizagem implícita em indivíduos com doença de Alzheimer, evidenciando que esse tipo de aprendizagem pode estar relativamente preservado mesmo na presença de prejuízos significativos na memória explícita. Essa constatação baseou-se na observação de que participantes com demência foram capazes de aprender informações funcionais por meio de técnicas como a recuperação espaçada ou exposição repetida, mesmo sem consciência declarativa da aprendizagem. Esses achados fornecem uma justificativa sólida para intervenções fonoaudiológicas que priorizem a comunicação funcional. Nesse sentido, a atuação precoce e preventiva do fonoaudiólogo é essencial, com foco em estratégias como triagens periódicas, reavaliações contínuas e uso de métodos como a aprendizagem sem erro, que evita reforçar respostas incorretas e tem se mostrado eficaz neste grupo. Dessa forma, visa-se aproveitar a memória procedural preservada e minimizar as demandas de aprendizagem declarativa^(16,18)^.

Embora não tenha como foco central a memória implícita, o estudo de Maziero et al.^(42)^ contribui para este campo de conhecimento ao utilizar o Teste de Gestão Implícita para investigar o desempenho de idosos com comprometimento cognitivo leve. Esse teste é uma medida que avalia a capacidade de compreensão de inferências durante atividades de leitura por meio de textos que contêm informações explícitas e implícitas, necessárias para a correta interpretação durante a leitura. Os achados ressaltam que, mesmo diante de déficits em memória episódica e em tarefas explícitas, aspectos do processamento implícito ainda podem exercer influência importante na compreensão textual. Esse tipo de evidência reforça a relevância de considerar estratégias terapêuticas baseadas em vias cognitivas alternativas e menos dependentes da recordação consciente em contextos de intervenção fonoaudiológica.

#### Linguagem Infantil

Temas relacionados à linguagem infantil foram recorrentes em 15^(19,21-23,27,30,33,35,38,40,43-45,52,53)^ estudos.

Dentro do campo da linguagem infantil, um dos temas mais abordados foi a Hipótese do Déficit Processual (PDH) no Transtorno do Desenvolvimento da Linguagem (TDL)^(19,21,23,30)^. Esta teoria foi proposta por Ullman e Pierpont^(56)^ para explicar os problemas linguísticos e não linguísticos observados em crianças com TDL. A PDH baseia-se no modelo declarativo/procedimental de aprendizagem da linguagem, segundo o qual as aquisições lexicais estão intimamente associadas à memória declarativa, enquanto a aprendizagem procedural apoia vários aspectos da gramática. Nessa perspectiva, a memória declarativa processa a ligação de representações conceituais, fonológicas e semânticas, enquanto a memória procedural, que é um dos vários sistemas envolvidos na aquisição implícita, sustenta aspectos da aprendizagem de regras e é particularmente importante para a aquisição e uso de habilidades que envolvem sequências, sejam elas abstratas, sensório-motoras ou cognitivas^(57)^.

Salienta-se que alguns estudos^(19,21,23,30)^ utilizam a nomenclatura ‘Comprometimento Específico de Linguagem’ (CEL) ou ‘Distúrbio Específico da Linguagem’ (DEL) , atualmente substituída por ‘Transtorno do Desenvolvimento da Linguagem’ (TDL), conforme as recomendações do projeto CATALISE^(58,59)^.

Tomblin et al.^(19)^ ao comparar adolescentes com TDL e com desenvolvimento típico (DT) usando o paradigma *Serial Reaction Time* (SRT), uma tarefa de aprendizagem implícita de sequências que mede o tempo que o participante leva para responder corretamente (por exemplo, apertar a tecla da caixa onde aparece uma imagem), viram que ambos os grupos melhoraram na tarefa de aprendizagem implícita (SRT), mas adolescentes com TDL apresentaram taxas de aprendizagem mais lentas. Essa diferença foi observada quando o comprometimento foi definido por gramática, mas não por vocabulário^(19,21,23,30)^.

Gabriel et al.^(23)^ realizaram estudo semelhante, porém com dois experimentos: o primeiro usando um teclado como modo de resposta e o segundo usando uma tela sensível ao toque, o que diminuiria as demandas motoras e cognitivas da tarefa. No primeiro experimento, com respostas via teclado, crianças com TDL foram mais lentas e cometeram mais erros; já no segundo, com respostas em tela sensível ao toque, essas diferenças desapareceram, sugerindo que as dificuldades anteriores podiam estar ligadas a exigências motoras e não a déficits de aprendizagem procedural. Portanto, esse estudo contraria o de Tomblin et al.^(19)^, sugerindo que os comprometimentos de linguagem em TDL não são sustentados por habilidades de aprendizagem procedural deficientes.

O estudo de Hsu e Bishop^(30)^ apoiou essa conclusão tendo utilizado o SRT e igualmente com uma tarefa de aprendizagem procedural motora que não envolvia a aprendizagem de relações sequenciais entre elementos discretos para testar crianças com TDL, crianças com desenvolvimento típico e idade correspondente, e crianças mais novas com desenvolvimento típico e gramática receptiva correspondente. Os resultados mostraram que as crianças com TDL tiveram o mesmo nível de desempenho das crianças com gramática correspondente, mas foram piores do que os controles com idade correspondente na aprendizagem de sequências motoras. No referido estudo, na tarefa de aprendizagem procedural motora, as crianças com TDL tiveram um desempenho comparável ao de crianças com idade correspondente e melhor do que os controles com gramática correspondente mais jovens. A partir deste resultado, pode-se concluir que os déficits em TDL estão mais ligados à aprendizagem de informações sequenciais do que a uma deficiência generalizada na aprendizagem procedural.

O estudo de Hedenius et al.^(21)^ examinou a consolidação e aprendizagem de sequências procedurais a longo prazo em crianças com TDL, utilizando a tarefa Alternating Serial Reaction Time (ASRT), que difere da SRT, pois as tentativas aleatórias são alternadas com tentativas seguindo a sequência fixa ao longo da tarefa. Os resultados indicaram prejuízos na consolidação e na aprendizagem de longo prazo, especialmente associados a déficits gramaticais, corroborando a ideia de que dificuldades na linguagem estão relacionadas a déficits específicos, e não generalizados, na aprendizagem procedural.

Além da investigação sobre os mecanismos cognitivos no TDL, alguns estudos se voltam para estratégias terapêuticas relacionadas ao tratamento desse transtorno. Uma das abordagens em destaque é o priming estrutural, utilizado tanto em estudos com crianças com TDL, quanto em pesquisas sobre afasia. Crianças com TDL demonstraram uma taxa de aprendizagem mais lenta com priming estrutural na produção de orações relativas com foco no sujeito e no objeto, em comparação com crianças com desenvolvimento típico^(19)^.

Nesse contexto, Wada et al.^(40)^ exploraram a viabilidade de combinar tarefas de priming implícito com reformulação explícita no ensino de orações relativas focadas no sujeito, mostrando que tais estratégias podem ser eficazes, embora provavelmente seja necessário um número maior de tentativas ou instruções adicionais para que crianças com TDL alcancem desempenho consistente.

Complementarmente, Baron e Arbel^(45)^ publicaram um tutorial que discute como diferentes características das intervenções fonoaudiológicas, como instruções, feedback e técnicas de elicitação, influenciam o envolvimento dos sistemas de aprendizagem implícita e explícita. Os autores destacam que, por conta do viés de desenvolvimento, a maioria das intervenções tende a envolver predominantemente o sistema explícito, tanto em crianças quanto em adultos. No entanto, a compreensão dos dois sistemas permite aos clínicos ajustar suas escolhas terapêuticas ao comportamento-alvo, posicionando a intervenção ao longo de um contínuo entre implícito e explícito. Um estudo aplicado, citado no próprio tutorial, demonstrou ganhos significativos em testes padronizados de gramática geral receptiva e expressiva e morfossintaxe expressiva a partir do uso combinado de técnicas implícitas e explícitas por um fonoaudiólogo.

O priming sintático também tem sido utilizado como estratégia para avaliar a aprendizagem implícita da sintaxe em crianças com TDL. O estudo de Calder et al.^(33)^ mostrou que, embora crianças com TDL apresentem efeitos imediatos de priming sintático, tais efeitos tendem a ser menos intensos e menos duradouros do que nos pares com desenvolvimento típico. Isso sugere que essas crianças têm dificuldade em aprender padrões gramaticais de forma implícita, extraindo menos informação útil de cada exposição linguística, o que dificulta a construção gradual de conhecimentos sintáticos mais complexos.

Nesse sentido, o estudo teórico de Montgomery et al.^(52)^ destaca que a aprendizagem de estruturas sintáticas depende de experiências repetidas de priming sintático. Assim, incluir atividades desse tipo no repertório de intervenção pode fortalecer o conhecimento sintático e promover melhorias na compreensão e produção de frases em crianças com dificuldades gramaticais.

Estudos sobre a aprendizagem implícita na dislexia do desenvolvimento (DD) também foram identificados. Uma das hipóteses teóricas mais consolidadas na área sugere que os déficits observados na DD estão associados a uma disfunção no sistema de memória procedural, envolvendo especialmente os circuitos córtico-cerebelares e/ou córtico-estriatais^(60)^.

Nesse contexto, Hedenius et al.^(21)^, em 2011, conduziram uma série de estudos para aprofundar a compreensão dos mecanismos de aprendizagem procedural em crianças com DD. Já em 2013^(27)^, os autores investigaram o desempenho dessas crianças não apenas durante a sessão inicial de prática, mas também em etapas posteriores, incluindo a consolidação noturna e a prática em um segundo dia. Os resultados sugeriram que os déficits de aprendizagem procedural mais pronunciados emergem após prática prolongada, indicando que as dificuldades associadas à DD podem se manifestar mais fortemente nos estágios posteriores da aprendizagem, o que reforça a hipótese de disfunção na memória procedural.

Em um estudo posterior, publicado em 2021, Hedenius et al.^(43)^, testaram se uma prática inicial mais longa poderia compensar as dificuldades de consolidação. Embora essa prática prolongada tenha sido implementada, os déficits na consolidação procedural persistiram, sugerindo que os problemas de aprendizagem observados anteriormente não se restringem à fase inicial, mas estão mais relacionados à consolidação da memória ao longo do tempo, especialmente durante o sono.

#### Distúrbios da Fluência e seus transtornos

Foram identificados três^(17,34,39)^ estudos sobre a gagueira. Dois deles utilizaram o Teste de Associação Implícita (TAI)^(34,39)^ para avaliar atitudes implícitas em relação a indivíduos que gaguejam. O TAI é um instrumento que avalia a latência de resposta na associação entre categorias-alvo e atributos valorativos, partindo do princípio de que associações mais fortemente consolidadas na memória implícita são processadas com maior rapidez e menor número de erros. O TAI permite, portanto, mensurar atitudes implícitas, que podem divergir das atitudes explicitamente declaradas pelos indivíduos^(61)^. O primeiro estudo, conduzido por Walden e Lesnar^(34)^, teve como objetivo investigar as atitudes implícitas e explícitas de jovens adultos com fluência típica em relação a pessoas que gaguejam. Para avaliar as atitudes explícitas, foram aplicadas escalas específicas e uma escala de desejabilidade social. Os resultados confirmaram a existência de estereótipos negativos associados à gagueira, com participantes demonstrando tanto atitudes negativas implícitas quanto explícitas.

O segundo estudo com o TAI, de Walden et al.^(39)^, teve foco semelhante, mas com fonoaudiólogos como participantes. Neste caso, as atitudes explícitas em relação a pessoas que gaguejam foram geralmente positivas, embora menos favoráveis do que em relação a pessoas que não gaguejam. As atitudes implícitas, no entanto, foram negativas. Esses achados destacam a importância de considerar os vieses implícitos na formação profissional, já que atitudes negativas não intencionais podem impactar o engajamento terapêutico e a empatia na relação clínico-paciente.

O terceiro estudo, de Anderson e Conture^(17)^, examinou o priming sintático em crianças que gaguejam (CWS) e crianças com desenvolvimento típico (CWNS). As CWS apresentaram tempos de reação mais lentos na ausência de frases de priming e demonstraram efeitos de priming sintático mais intensos, sugerindo uma maior dependência de pistas estruturais externas. Esses achados indicam que a gagueira pode estar relacionada a dificuldades no acesso rápido e eficiente a representações sintáticas, o que pode contribuir para a disfluência. Tais evidências apontam para o potencial terapêutico de estratégias que explorem a estruturação implícita da linguagem.

### Audiologia

Foram identificados quatro estudos na área da audiologia^(25,29,41,51)^. Dois^(29,51)^ deles utilizaram o Teste de Associação Implícita (TAI).

O estudo de Beadle et al.^(51)^ investigou as percepções de idosos e jovens adultos sobre pessoas das mesmas faixas etárias que utilizam aparelhos auditivos. Para isso, os participantes completaram dois testes de associação implícita: um com imagens de adultos mais velhos e outro com adultos mais jovens, ambos com e sem aparelhos auditivos. Também avaliaram a idade, atratividade e inteligência das pessoas retratadas. Enquanto as tarefas de julgamento explícito indicaram atitudes neutras em relação aos usuários de aparelhos auditivos, os resultados do TAI sugerem a presença de atitudes implícitas negativas, já que os participantes responderam mais rapidamente e com maior precisão quando imagens de usuários de aparelhos auditivos foram associadas a palavras negativas, em comparação com palavras positivas.

Já o estudo de Moring et al.^(29)^ avaliou a viabilidade do uso do TAI para medir atitudes relacionadas ao zumbido, além de investigar se instrumentos tradicionais, como o Tinnitus Handicap Inventory (THI), funcionariam como estímulos de priming. Embora as palavras relacionadas ao zumbido não tenham gerado associações implícitas fortes, e as pontuações do TAI não tenham previsto os escores no THI, os autores observaram que o preenchimento prévio de questionários explícitos pode influenciar negativamente os resultados do TAI, ao ativar um viés negativo nas respostas. Assim, o estudo sugere que estímulos auditivos reais (como sons de zumbido) podem ser mais eficazes do que palavras escritas na ativação de estruturas implícitas relacionadas ao zumbido.

O estudo de Evans^(41)^ aborda preconceitos e vieses implícitos na prática audiológica, incluindo a sub-representação de profissionais negros, o viés socioeconômico e linguístico, e o audismo. Destaca como esses fatores afetam a forma como as informações são compartilhadas com famílias, e propõe uma abordagem afirmativa e culturalmente sensível na comunicação clínica. Propõe a reflexão sobre o currículo da pós-graduação e em como o currículo pode refletir com mais precisão as comunidades que atendem.

Além dos estudos que investigam atitudes implícitas na audiologia, foi identificado um artigo que, embora não tenha como foco central a aprendizagem implícita, aborda sua atuação no processamento da linguagem em condições auditivas adversas. O estudo de Rönnberg et al.^(25)^ propõe uma atualização do modelo Ease of Language Understanding (ELU), que descreve como a memória de trabalho interage com mecanismos de processamento implícito e explícito na compreensão da fala. O modelo sugere que o processamento implícito rápido é essencial para o reconhecimento automático da fala quando o sinal auditivo é claro, enquanto o processamento explícito (mais lento e dependente da memória de trabalho) é recrutado quando há distorções no sinal, como em situações com ruído ou uso de aparelhos auditivos. Embora o foco principal seja a memória de trabalho, o reconhecimento do papel de mecanismos implícitos nesse modelo amplia a compreensão sobre como o processamento auditivo interage com diferentes sistemas de aprendizagem, especialmente em populações com perda auditiva.

### Voz

Os dois^(47,55)^ estudos encontrados na área de voz abordam preconceitos implícitos na prática clínica vocal. O primeiro, de Norotsky et al.^(47)^, investigou o papel do viés racial implícito nas avaliações perceptivo-auditivas de vozes disfônicas, com o objetivo de verificar se a raça do falante influenciava as avaliações feitas por ouvintes novatos. Estudantes de pós-graduação em Fonoaudiologia ouviram gravações de falantes negros e brancos com distúrbios vocais, avaliaram a voz por meio de uma escala analógica visual de 100 unidades e, em seguida, realizaram o TAI para medir seu viés racial. Os resultados sugeriram a presença de um viés minimizador nas avaliações de falantes negros, indicando que pacientes negros com distúrbios vocais podem receber avaliações menos severas, o que pode comprometer o diagnóstico e tratamento adequado.

Esses achados são aprofundados no estudo de Morton-Jones e Sund^(54)^, que discutem de forma mais ampla o impacto do preconceito implícito na prática clínica em voz. O artigo ressalta que viés inconsciente e ausência de práticas culturalmente responsivas podem comprometer a qualidade do cuidado fonoaudiológico e até afastar pacientes da busca por tratamento vocal. Assim, os autores reforçam a necessidade de que fonoaudiólogos e equipes interdisciplinares desenvolvam estratégias conscientes de enfrentamento aos preconceitos implícitos, promovendo atendimentos mais éticos, equitativos e inclusivos.

#### Sobre a avaliação da qualidade metodológica

Em relação ao nível de evidência dos estudos, observa-se que a maioria dos estudos se enquadra no nível 3 (36,0%), indicando predominância de evidências derivadas de estudos observacionais, estudos de coorte ou caso-controle, enquanto apenas 5,1% alcançam o nível 2, correspondendo a evidências de ensaios clínicos randomizados bem conduzidos. Os níveis 4 e 5, que representam estudos de séries de casos, relatos de casos ou opiniões de especialistas, correspondem a 3,3% e 25,6% dos estudos, respectivamente. Esses achados refletem a heterogeneidade metodológica da literatura sobre memória implícita em Fonoaudiologia e sugerem que, embora existam estudos experimentais, grande parte das evidências ainda é baseada em delineamentos observacionais ou relatos, o que reforça a necessidade de investigações com delineamentos mais robustos e padronização metodológica, a fim de consolidar a base de conhecimento e subsidiar práticas clínicas mais fundamentadas em evidências.

### Limitações e contribuições para o futuro

Como limitação, observa-se que, do ponto de vista conceitual, há ausência de padronização de termos e certa ambiguidade na definição de memória implícita nos estudos incluídos. Em relação aos instrumentos de avaliação, a diversidade de tarefas utilizadas e a falta de padronização podem dificultar comparações entre os achados. Por fim, quanto à generalização dos resultados, os estudos se concentram principalmente em algumas áreas da Fonoaudiologia, como linguagem, audiologia e voz, sugerindo a possibilidade de investigação em outros contextos clínicos e subáreas do campo.

Uma das contribuições desta revisão é apontar aos especialistas as lacunas existentes nas demais áreas da fonoaudiologia como potenciais campos de investigações com aplicabilidade clínica.

## CONCLUSÃO

A produção científica internacional em Fonoaudiologia tem abordado a memória implícita de forma ainda incipiente e heterogênea, concentrando-se principalmente nas áreas de linguagem, audiologia e voz. Observa-se predomínio de estudos que exploram a aprendizagem implícita como recurso terapêutico, o uso de tarefas implícitas para avaliação e caracterização de transtornos, bem como investigações sobre vieses e percepções implícitas na prática fonoaudiológica. Apesar de evidenciar o potencial da memória implícita para ampliar estratégias avaliativas e interventivas, a literatura apresenta limitações conceituais e metodológicas, incluindo a ausência de padronização terminológica e a diversidade de delineamentos e instrumentos utilizados. Esses achados indicam a necessidade de investigações futuras mais sistematizadas, com maior clareza conceitual e metodológica, a fim de consolidar o papel da memória implícita no campo da Fonoaudiologia e subsidiar práticas clínicas baseadas em evidências.
